# Early life vitamin D status and asthma and wheeze: a systematic review and meta-analysis

**DOI:** 10.1186/s12890-018-0679-4

**Published:** 2018-07-20

**Authors:** Song-Ying Shen, Wan-Qing Xiao, Jin-Hua Lu, Ming-Yang Yuan, Jian-Rong He, Hui-Min Xia, Xiu Qiu, Kar Keung Cheng, Kin Bong Hubert Lam

**Affiliations:** 1 Division of Birth Cohort Study, Guangzhou Women and Children’s Medical Center, Guangzhou Medical University, 9 Junsui Road, Zhujiang Newtown, Tianhe District, Guangzhou, 510623 China; 2 Department of Pediatric Surgery, Guangzhou Women and Children’s Medical Center, Guangzhou Medical University, Guangzhou, China; 3 Institute of Applied Health Research, University of Birmingham, Birmingham, UK; 4 Clinical Trial Service Unit and Epidemiological Studies Unit, Nuffield Department of Population Health, University of Oxford, Oxford, UK

**Keywords:** Vitamin D, Asthma, Wheeze, Pregnancy, Fetal blood, Systematic review, Meta-analysis

## Abstract

**Background:**

Vitamin D deficiency has been linked to an increased risk of asthma. This study aimed to quantify the effect of early life vitamin D status on asthma and wheeze later in life.

**Methods:**

PubMed, Embase, CINAHL, and CNKI databases, the Cochrane Central Register of Controlled Trials, and Google Scholar were searched up to July 2017. We included randomized controlled trials (RCTs) and cohort studies with vitamin D level in blood (maternal or cord or infant) or intake (maternal intake during pregnancy or infant intake) and asthma and/or wheeze. Two reviewers independently extracted data. Fixed- and random-effects models were used to summarize the risk estimates of comparisons between highest vs. lowest vitamin D categories.

**Results:**

Of the 1485 studies identified, three RCTs and 33 cohort studies were included. We did not include the RCTs (1619 participants) in the meta-analysis as the comparators and outcome definitions were heterogenous. Three RCTs reported a non-statistically significant effect of vitamin D supplementation during pregnancy on offspring wheeze/asthma at 3 years of age. Pooled estimates of cohort studies suggest no association between antenatal blood vitamin D levels or vitamin D intake and offspring asthma assessed either > 5 years or ≤ 5 years. The estimate for blood vitamin D remained unchanged when two studies assessing asthma in adulthood were excluded, but a significant inverse association emerged between vitamin D intake and childhood asthma. We found no association between antenatal vitamin D level and wheeze. On the other hand, vitamin D intake during pregnancy may have a protective effect against wheeze.

**Conclusions:**

The pooled estimates from cohort studies show no association between antenatal blood vitamin D level and asthma/wheeze in later life. Whereas, the pooled estimates from cohort studies suggest that antenatal vitamin D intake may have an effect on childhood asthma > 5 years or childhood wheeze. The inconsistent results from studies assessing vitamin D either in blood or intake may be explained by previously reported non-linear association between blood vitamin D_3_ and childhood asthma. Further trials with enough power and longer follow-up time should be conducted to confirm the results.

**Electronic supplementary material:**

The online version of this article (10.1186/s12890-018-0679-4) contains supplementary material, which is available to authorized users.

## Background

Asthma is an important cause of disability and a major worldwide public health concern. The prevalence of asthma in developed countries rose rapidly in the 1960s and has remained high since [1]. More recently, the same rising trend has been observed in developing countries [2]. While asthma affects people of all ages, the surge among children has been most marked [3].

Among the environmental factors that contribute to asthma, vitamin D status has generated increasing interest for the vitamin’s purported immunomodulatory properties.

Clinically, serum calcifediol [25(OH)D] is used as a marker of vitamin D level [4]. In various populations calcifediol levels in cord blood are strongly correlated with maternal levels during pregnancy [5], with maternal calcifediol as the source of the fetal vitamin D pool [6]. Vitamin D deficiency in pregnant women and infants is common worldwide, including both developed and developing countries, ranging from 45 to 90% in pregnant women and 61–94% in infants [5, 7–9]. Based on growing epidemiological evidence, vitamin D deficiency has been linked to an increased risk of respiratory infections and asthma [10]. However, it is still unclear if and to what extent antenatal or early postnatal vitamin D deficiency would affect the development of wheeze or asthma later in life. Previous reviews on vitamin D supplementation during pregnancy have given conflicting messages [11–13].

Given that there have been further published studies (including trials), we undertook a systematic review aiming to address whether antenatal or early postnatal vitamin D status (including intake and blood level, both maternal and in infant) has any impact on the risk of developing asthma and wheeze later in life.

## Methods

We followed the Meta-analysis of Observational Studies in Epidemiology (MOOSE) [14] and the Preferred Reporting Items for Systematic Reviews and Meta-Analyses (PRISMA) guidelines [15] when conducting and presenting this systematic review and meta-analysis. PRISMA and MOOSE checklist can be found in Additional file 1: Supplement 1. The review protocol was registered previously with the International Prospective Register of Systematic Reviews (PROSPERO Registration No. CRD42013005559) [16]. The initial protocol considered asthma/wheeze, allergic rhinitis, atopic dermatitis (eczema), food allergy and atopic sensitization, but here we focus on asthma and wheeze.

### Data sources and searches

We conducted a systematic literature search using PubMed, Embase, CINAHL, and CNKI (in Chinese) databases and Google Scholar for studies published up to 19th July 2017 and the Cochrane Central Register of Controlled Trials that reported data on antenatal or early postnatal vitamin D status or intervention and asthma and wheezing in children or adults. Details of the search strategy are provided in Additional file 1: Supplement 2.

### Study selection

The present systematic review focused on the effect of vitamin D level in blood (maternal or cord or infant) and intake (maternal intake during pregnancy or infant intake) on asthma and wheeze. We included randomized controlled trials (RCTs), quasi-randomized controlled trials, non-randomized controlled trials, prospective and retrospective cohort studies that measured maternal vitamin D status during any trimester of pregnancy or cord blood or offspring vitamin D status during infancy, and having asthma or wheeze during childhood or adulthood as outcomes, which (i) were diagnosed by doctors (including parental or self-reports), or (ii) required the use of asthma medication, or (iii) as assessed by the International Study of Asthma and Allergies in Children (ISAAC) questionnaire (Additional file 1: Supplement 3). Two reviewers (SS and WX) independently screened the titles and abstracts and identified potentially relevant publications according to the selection criteria, of which full text was obtained and read (online Additional file 1: Supplement 4). The reference lists of all papers of interest were scrutinized to obtain other relevant articles. Disagreements over inclusion were resolved through consensus, and where necessary, a third reviewer (JL) was involved.

### Data extraction and risk of Bias assessment

The same reviewers independently extracted data using a standard data extraction form. We collected data on the number of subjects with and without asthma or wheeze in the antenatal or early postnatal vitamin D exposed/intervention and non-exposed/control groups; risk estimates (crude and/or adjusted odds ratios [ORs], relative risks [RRs] and hazard ratios [HRs]) and the corresponding 95% confidence intervals (CIs) at any available age end point. Furthermore, we recorded information on the population, geographical location, inclusion and exclusion criteria, interval of follow up, exposure measurement, outcome definition, measurement and window of assessment, and confounders (more details in Additional file 1: Supplement 5). Study authors were contacted when missing data was an issue. Disagreements on data extraction were checked against the original articles and/or resolved by a third reviewer. The data extracted were entered into Review Manager software and were double checked by two reviewers. Risk of bias was assessed using the tool recommended by the Cochrane Collaboration [17, 18]. Two reviewers independently rated the risk of bias for each study as high, low, or unclear (‘Definitely yes’ in the tool was considered as ‘low’ risk, ‘Definitely no’ as ‘high’ risk, ‘unclear’ comprised ‘probably yes’ and ‘probably no’ as specified in the tools [18]). Disagreements were resolved by discussion or by involving a third reviewer. Each cohort study was classified into one of the following categories: high (more than two criteria not applied/met); moderate (one or two criteria not applied/met or unclear); and low risk of bias (all criteria applied/met).

### Data synthesis and analysis

We pooled the original risk estimates as reported (most being ORs) and used adjusted risk estimates where available. When only frequency distributions were provided, we calculated unadjusted ORs and their 95% CIs from the outcome distribution of exposed and non-exposed groups. Where outcome assessment was done in multiple time points in a study, we chose the one with the longest follow-up period or the time point when the age of the participants was comparable to other studies.

We pooled the risk estimates from each study that compared the risk in the highest vitamin D concentration or intake category with the lowest (which is the referent category), and also the risk in vitamin D sufficiency (≥75 nmol/L [4]) with deficiency (< 50 nmol/L according to the Institute of Medicine (IOM) definition [19]). As it could be difficult to diagnose asthma in children below 5 years of age, we stratified the studies on asthma according to the age of outcome assessment (> 5 years and ≤ 5 years). For exploring the association between blood vitamin D status and childhood asthma, a sensitivity analysis was performed by excluding the studies assessing the outcome in adulthood. We contacted the authors of the studies (*n* = 8) that had different cut-off values, and asked them to re-analyze their data according to the above categorization scheme. Of the eight authors contacted, four responded and provided additional data [20–23].

Both fixed-effects and random-effects models were used to summarize the effect sizes. Heterogeneity was evaluated by using the Q (*P* > 0.10 in the Chi^2^ test for low heterogeneity) and I^2^ statistics (moderate heterogeneity for I^2^ > 30% and considerable heterogeneity for ≥75%). We presented pooled ORs from random-effect models when considerable heterogeneity was observed. Stratified analyses by time window of vitamin D and outcome assessment (for wheeze only), latitude, geographical region, adjustment for family history of allergic disease, seasonality, and smoking status, methods of exposure and outcome ascertainment, and risk of bias were performed to explore the sources of heterogeneity. Funnel plots and Egger’s test were used to explore the possibility of publication bias. Number needed to treat for preventing asthma by increasing from low to high levels of vitamin D or via supplementation was estimated by the baseline asthma prevalence estimates and pooled ORs [24]. Statistical analyses were performed with Review Manager software version 5.3 [25] and STATA version 13.

## Results

### Search results

**Fig. 1:**
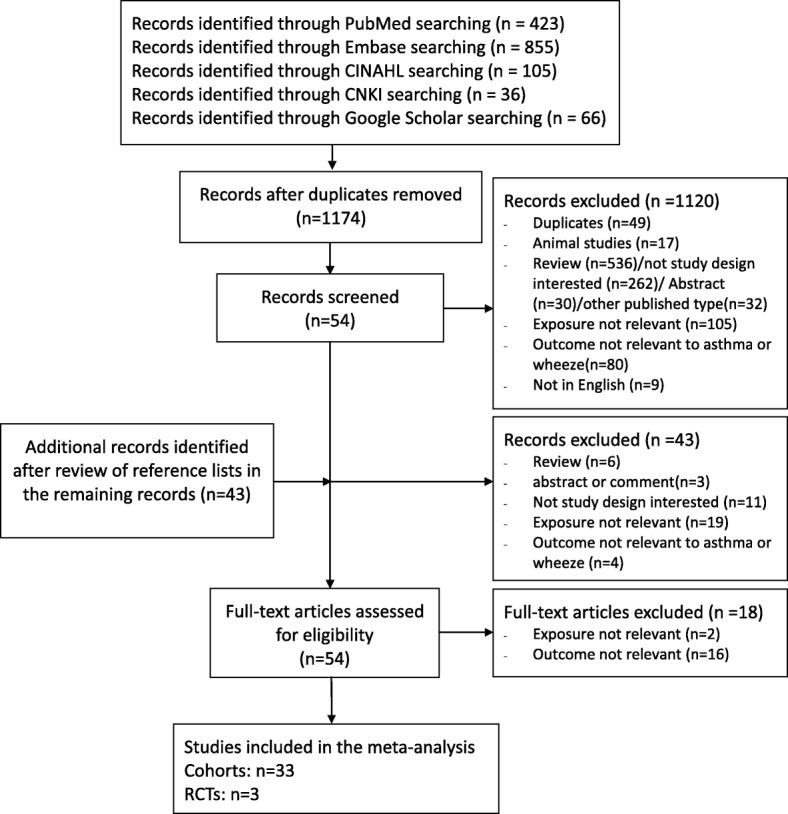
Summary of literature search and study selection

We have identified 423 potentially relevant publications from PubMed, 855 from Embase, 36 from CNKI database, 105 from CINAHL and 66 from Google Scholar. After excluding duplicates and publication that did not meet the inclusion criteria, three RCTs [26–28] and 33 cohort studies [20–23, 29–57] were included (Fig. 1).

### Study characteristics

The main characteristics of the eligible RCTs, cohort studies and those studies excluded during full text screening are shown in Tables 1 and 2 and Additional file 1: Table S1, respectively. Two of the three RCTs were single-center trials and recruited low-risk pregnant women. The other was a multicenter trial enrolling pregnant women who had history of allergic disease. The dose of vitamin D administration and outcome definition varied across the three trials (1619 participants) (Table 1). The median ages of outcome assessment in cohort studies that reported association between blood vitamin D level and asthma, vitamin D intake and asthma, blood vitamin D and wheeze, and vitamin D intake and wheeze were 6 years (range 1.5–25), 5 (1.5–31), 5 (1–10), and 3 (1–10), respectively. Only two studies assessed the outcome in adulthood [36, 57]. Of the cohort studies, 26 studies reported data on asthma and 17 on wheeze. Nineteen of these 26 asthma studies measured vitamin D levels (31,940 participants from 13 cohorts were included in the meta-analysis); the rest assessed dietary/supplement intake (41,952 participants). A variety of operational definitions of asthma were used, including parental reports of doctor-diagnosed asthma (*n* = 7), parental reports of asthma medication use (*n* = 1), a combination of both (*n* = 2), parental reports of doctor-diagnosed asthma with wheezing symptom (*n* = 4), parental reports of doctor-diagnosed asthma and wheezing and/or asthma medication use (*n* = 5) and doctor-diagnosed asthma or asthma medication use by medical record review (*n* = 5). Two studies did not report the definition of asthma. All 17 studies on wheeze assessed maternal vitamin D status during pregnancy, with 12 measuring blood vitamin D levels (8123 participants from 8 cohorts were meta-analyzed) and 6 recorded intake (5678 participants). Most wheeze studies defined outcome using parental reports of wheezing symptoms in the past 12 months (Table 2).

**Table 1:** Characteristics of the randomized controlled trials

Reference	Design	Patients characteristics	Intervention (Number of participants)	Comparator (Number of participants)	Outcomes assessment and definition (Age of outcome assessment)
Goldring, 2013 [26]	Single-center non-blinded non-placebo RCT	Pregnant women in London (51°N) (27 weeks gestation, Asian, Middle Eastern, Black and White) without sarcoidosis, osteomalacia, renal dysfunction or tuberculosis	1. Daily vitamin D (800 IU ergocalciferol until delivery) (n = 60) 2. Bolus vitamin D (a single oral dose of 200,000 IU cholecalciferol (*n* = 60)	No treatment (*n* = 60)	1.Wheeze ever (ISAAC, at age 3) 2. Recurrent wheezing (≥2 episodes of reported wheezing since birth, at age 3) 3. Wheeze in the year prior to assessment (ISAAC, at age 3) 4. Wheeze with a positive asthma predictive index (loose criteria, at age 3)
Chawes, 2016 [27]	Single-center double-blinded RCT	Pregnant women in Denmark (55°43’N) (≤26 gestational week) without any endocrine, cardiovascular, or nephrological disorders; or vitamin D3 (cholecalciferol) intake more than 600 IU/d	Daily dose of 2400 IU vitamin D3 supplementation (24 gestational week to 1 week postpartum) (*n* = 315)	Matching placebo tablets (Camette A/S) (24 gestational week to 1 week postpartum) (*n* = 308)	1.Persistent wheeze (diagnosed according to a previously validated quantitative algorithm, from birth to age 3 years) 2.Asthma (doctor diagnosed in children fulfilling the persistent wheeze criteria at age 3)
Litonjua, 2016 [28]	Multicenterdouble-blinded placebo RCT	Pregnant women in Boston (52°58’N), Washington (38°53’N), San Diego (32°43’ N) (age 18–39,10–18 gestational week, nonsmoker, English or Spanish speaking, who or whose partner had a history of allergic disease, with intent to participate for 4 years)	Daily 4000 IU of vitamin D plus a multivitamin with 400 IU of vitamin D (*n* = 440)	Placebo (daily placebo pill plus a multivitamin with 400 IU of vitamin D) (*n* = 436)	Asthma or recurrent wheeze in first 3 y of life (parental report, every 3 months)

**Table 2:** Characteristics of cohort studies investigating the relationship between antenatal and early postnatal vitamin D exposure and asthma/wheeze

Reference	Location (latitude)	Exposure	Exposure assessment (vitamin D form)	Period of exposure assessment	Median (IQR) of vitamin D status	Outcome assessment and definition (Age outcome assessment), sample size
Pike, 2012 * [22]	Southampton, UK (50°54’N)	Maternal blood	Radioimmunoassay (Total 25(OH)D)	34 wk. gestation	59.0 (IQR, 40.5–84.9) nmol/L	Asthma (6y): Maternal report of doctor diagnosed asthma, *n* = 860 Wheeze (6, 12, 24, 36mo, 6y): Maternal report of wheezing symptom in the last 12 months (ISAAC questionnaire), n = 860
De Jongh, 2014* [29]	Southampton, UK, (50°54’N)	Maternal blood	Radioimmunoassay (Total 25(OH)D)	34 wk. gestation	59.0 (IQR, 40.6–84.3) nmol/L	Wheeze (0–6, 6–12, 12-24mo): Parental report of one or more episodes of chest wheezing/whistling, *n* = 856
Morales, 2012 [30]	4 study areas in Spain (39°N, 39°N, 41°N, 44°N)	Maternal blood	HPLC (25(OH)D3)	77%:12–23 wk.22%:< 12 wk.1%: 24–36 wk	73.8 (IQR,56.3–92.8) nmol/L	Asthma (4-6y): Mother report of medication for asthma or wheezing, *n* = 1233 Wheeze (1, 4y): parental report of wheezing symptom in the last 12 months (questionnaire), n = 1233
Gale, 2008 [20]	Southampton, UK (50°54’N)	Maternal blood	Radioimmunoassay (Total 25(OH)D)	Late pregnancy	50 (IQR,30 to 75.3) nmol/L	Asthma(9y): Unclear, *n* = 178
Zosky, 2014 [31]	Perth, Australia (31°57’S)	Maternal blood	ELISA (Total 25(OH)D)	16–20 wk. gestation; mean 18 wk	NA	Current asthma (6, 14y): maternal report of doctor diagnosis of asthma at any time, with wheeze and use of any asthma medication in the past 12 months. *n* = 291 Current wheeze (6, 14y): questionnaire, n = 291
Maslova, 2014 [32]	Nationwide, Denmark (55.4°N)	Maternal blood (prediction score)	LC-MS/MS (Total 25(OH)D) (use of prediction model)	25 wk. gestation in 1497 pregnant women	58.7(IQR,49.2–69.0) nmol/L	Asthma (18 months): parental report of doctor-diagnosed asthma, *n* = 24,662 Current asthma (7y): doctor diagnosis of asthma and parental report of wheeze in the past 12 months, *n* = 21,194
Wills, 2013 [33]	South West of England (50–51.5°N)	Maternal blood	HPLC-MS/MS (Total 25(OH)D: sum of 25(OH)D2 and 25(OH)D3)	34 wk. gestation (Translation by model)	NA	Asthma (7.5y): Maternal report of doctor-diagnosis of asthma plus report of wheeze or asthma (questionnaire), *n* = 4648 Wheeze (7.5y): Maternal report of wheezing symptom in the last 12 months, *n* = 4696
Magnus, 2013 [34]	Nationwide, Norway (57°54′ -70°55’ N)	Maternal blood	LC-MS/MS (Total 25(OH)D: sum of 25(OH)D2 and 25(OH)D3)	Approximately 18 wk. gestation	73.7 (SD 23.7) nmol/L	Asthma (3y): Maternal report of asthma plus name of medication for asthma (questionnaire), *n* = 1246
Chiu, 2015 [35]	Taiwan (25°N)	maternal blood, cord blood	electrochemiluminescence-based assay	before delivery, cord blood	Maternal blood: 58.3 (SD 19.3) nmol/L Cord blood: 59.5 (SD 23.8) nmol/L	Asthma (at 4y): ever having asthma, with the occurrence of recurrent wheeze in the last 12 months, or current use of asthma medication (only the results of maternal blood and children blood at 4y were reported), *n* = 119
Hansen, 2015 [36]	Aarhus, Denmark (56°9’ N)	maternal blood	LC-MS/MS	third trimester of pregnancy	76 (IQR 57) nmol/L	Asthma: medication use (at 25y), medical record hospitalizations (at 25y), medical record self-reported lifetime doctor’s diagnosis of asthma(at 20y) Current asthma medication use (at 20y), *n* = 850
Gazibara, 2016 [37]	Rotterdam, the Netherlands (51°55’ N)	maternal blood cord blood	LC-MS/MS	mid-gestation delivery	Maternal blood: 62.8 (range 2.3–193.2) nmol/L Cord blood: 40.2 (range 0.1–144.9) nmol/L	Asthma (6 y): parent reported physician-diagnosed, *n* = 158 Persistent wheezing(1, 2, 3, 4 and 6 y): at least 1 wheezing episode in the first 3 years of life and 1 episode of wheezing at 4 or 6 years of age (ISAAC), *n* = 255
Baiz, 2014^#^ [38]	Poitiers (46°35’N), Nancy (48°41’N), France	Cord blood	Immunochemiluminescent immunoassay (Total 25(OH)D)	Delivery	44.5 (IQR 37.8) nmol/L	Asthma (5y): parental report of a doctor’s diagnosis of asthma plus either 1 or more attacks of wheeze or use of asthma medication in the last 12 month, *n* = 239 Wheeze (1, 2, 3, 5 y): parental report of wheezing in the past 12 months (ISAAC phase I questionnaire), n = 239
Rothers, 2011 [23]	Tucson, Arizona, USA (32°N)	Cord blood	LC-MS/MS (Total 25(OH)D: sum of 25(OH)D2 and 25(OH)D3)	Delivery	64 (IQR 49–81) nmol/L	Asthma (1, 2, 3, 5y): Parental report of doctor diagnosed asthma, *n* = 194
Camargo, 2011 ^#^ [39]	Wellington (41°S), Christchurch (43°S), New Zealand	Cord blood	Automated chemiluminescent immunoassay (Total 25(OH)D)	Delivery	44 (IQR, 29–78) nmol/L	Asthma (3,15 mo, 2, 3, 4, 5y): Any previous report of doctor-diagnosed asthma by 5y plus either a history of inhaler use or wheeze since 4y, *n* = 181 Wheeze (15mo, 2, 3, 4, 5y): Parental report of wheeze at any time before, *n* = 533
Stelmach, 2015 [40]	8 different regions of Poland (54°22 N)	Cord blood	HPLC (Total 25(OH)D)	Delivery	15.8 (IQR, 10.4, 21.3) nmol/L	Viral-induced wheezing (2 y): wheeze ever appearing during infection. Multi-triggered wheezing (2 y): wheezing triggered by two or more factors (e.g., viral infection, weather, activity) (ISAAC based questionnaire with information from medical chart), *n* = 190
Chawes, 2014 [41]	Copenhagen, Denmark (55°43’N)	Cord blood	LC-MS/MS (Total 25(OH)D: sum of 25(OH)D2 and 25(OH)D3)	Delivery	47.6 (range, 10–145) nmol/L	Asthma (7y): Doctor diagnosed, *n* = 257
Jones, 2012 [21]	Perth, Australia (31°57’S)	1. Cord blood 2. Maternal intake (total/diet/ supplement)	1. LC-MS/MS (25(OH)D3) 2. FFQ	Last trimester of pregnancy	Blood: 58.4 (SD, 24.1) nmol/L Intake: 200 (SD, 248) IU/d	Wheeze (12mo): Unclear, *n* = 231
Junge, 2015 [42]	Leipzig, Germany (51.4 N)	Cord blood Infant blood-one year, two year	HPLC-MS/MS (25(OH)D 3)	Delivery Infant-one year Infant -two year	Cord blood: 27.4 (IRQ, 17.5, 43.5) nmol/L One year: 83.0 (IRQ, 70.5, 97.7) nmol/L two year: 55.6 (IRQ, 42.2, 70.7) nmol/L	Wheezing ever, Wheezing recurrent (0–36 months;12–36 months;24–36 month) Unclear, *n* = 367
Palmer, 2015 ^#^ [43]	Adelaide, Australia (34°51’ S)	Cord blood	LC-MS/MS (25(OH)D3)	Delivery	55.9 (SD, 28.4) nmol/L	Asthma (1, 3 y): as a history of 3 or more episodes of wheeze with the episodes less than 6 weeks apart and/or daily use of asthma medication (medical review), *n* = 8123
Visness, 2015 ^#^ [44]	URECA: (Baltimore 39°17′N, Boston 52°58’N, New York40°44‘N, and St Louis 38°4’N) COAST (Madison, Wisconsin 43°05‘N)	Cord blood	Unclear	Delivery	URECA cohort:20.1 (rang, 4.2–54.6) nmol/L COAST cohort: 21.1 (range, 4.0–77.7) nmol/L	Recurrent wheeze (URECA cohort, every 3 months): at least two episodes of wheezing during the first three years of life with at least one episode during the third year (parent reported), *n* = 435 . Any wheeze (COAST cohort, annual): unclear definition (phone contacts & questionnaire), n = 258 Current asthma (COAST cohort, 6y): doctor diagnosed, *n* = 258.
Wolsk, 2017 [45]	Boston (52°58’N), Washington (38°53’N), San Diego (32°43’ N)	Maternal blood	chemiluminescence immunoassay	weeks 10–18 of gestation	56.3 (SD 25.3) nmol/L	Asthma or recurrent wheeze in first 3 y of life (parental report, every 3 months), *n* = 712
Hollams, 2016# [46]	Western Australia(31°57’S)	Vitamin D over the first decade of life	LC-MS/MS (25(OH)D3)	Birth, 6 months, 1 year, 2 years, 3 years, 4 years, 10 years	Birth:26.2 (IQR 20.3, 36.9) nmol/L 6 months: 68.7 (IQR 55.4, 80.5) nmol/L 1 year: 62.1 (IQR 50.1 to 74.6) nmol/L 2 years: 58.6 (IQR 49.6, 68.0) nmol/L 3 years: 58.6 (IQR 46.5, 67.5) nmol/L 4 years: 57.5 (IQR 47.4 to 67.6) nmol/L 5 years: 89.0 (IQR 71.5, 97.4) nmol/L 10 years:76.2 (IQR 64.1, 87.6) nmol/L	Current asthma (from age 3 to 10 years): parent reported wheeze in the last 12 months in children who had ever been given a diagnosis of asthma by a doctor. *n* = 216 Wheeze (from age 1 to 10 years): parent reported wheezing symptom in the last 12 months, *n* = 235
Wegienka, 2015# [47]	Detroit, Michigan (42°19’N)	maternal blood cord blood infant blood at 2 age	HPLC (Total 25(OH)D: sum of 25(OH)D2 and 25(OH)D3)	pregnancy, delivery (cord blood) and at age 2 years	Prenatal; 58 (SD 29.3) nmol/L Cord: 27 (18.5) nmol/L 2 years: 59.5 (20.3) nmol/L	Asthma (3–6 years): parent-reported diagnosed, *n* = 635
Miyake, 2010 [48]	Neyagawa, Japan (34°45′58”N)	Maternal intake (diet)	FFQ	During pregnancy (5~ 39 wk)	6.2 (SD 3.7) μg/d	Wheeze (16-24mo): Maternal report wheezing symptom in the last 12 months (ISAAC phase-I questionnaire), *n* = 763
Devereux, 2007** [49]	Grampian region, Aberdeen, UK (57°N)	Maternal intake (total)	FFQ	32 wk. gestation	128 (IQR, 99–170) IU/d	Asthma (5y): Maternal report of doctor-diagnosed asthma Wheeze (2, 5y): Maternal report of wheezing symptom in the last 12 months (ISAAC questionnaire), *n* = 1212
Allan, 2015** [50]	Grampian region, Aberdeen, UK (57°N)	Maternal intake (total)	FFQ	32 wk. gestation	3.60 (95% CI 3.50–3.71) μg/d	Asthma (10y): 1. Parental report of doctor-diagnosed asthma 2. Current asthma: asthma and wheeze in the previous year, *n* = 919 Wheeze (10y): Parental report of wheeze in the past 12 months (ISAAC based questionnaire), *n* = 924
Camargo, 2007 [51]	Eastern Massachusetts, USA (41°10’N to 42°53’N)	Maternal intake (total/diet/ supplement)	FFQ	During the first and second trimesters	548 (SD 167) IU/d	Wheeze (1, 2, 3y): Parental report of recurrent wheeze as ≥2 wheezing attacks (summed from the 1-, 2-, and 3-y annual questionnaires) in children with a personal diagnosis of eczema or parental history of asthma, *n* = 1194
Maslova, 2013 [52]	Nationwide, Denmark (55.4°N)	Maternal intake (total/diet/ supplement)	FFQ	25wk (mid-pregnancy)	11.7(5th -95th percentile, 3.0–19.4) μg/d	Asthma (18mo): Maternal report of doctor diagnosed asthma (questionnaire), *n* = 28,421 Asthma (7y): Combination of lifetime doctor diagnosis of asthma and wheezing symptoms in the past 12 months (ISAAC questionnaire), *n* = 24,382
Erkkola, 2009 [53]	Turku (60°27’N), Oulu (65° 0′46”N), Tampere (61° 5’ N), Finland	Maternal intake (total/diet/supplement)	FFQ	8th month of pregnancy	6.5 (SD 3.8) μg/d	Asthma (5y): Maternal report of doctor diagnosed asthma plus medication for wheezing or asthma (questionnaire), *n* = 1669
Miyake, 2014 [54]	Kyushu Island, Okinawa Prefecture, Japan (33°N, 26°33’N)	Maternal intake (diet)	FFQ	During pregnancy (during the preceding month among women at the 5 and 39 wk. gestation)	5.8 (SD 3.0) μg/d	Asthma (23–29 mo): physician-diagnosed asthma, *n* = 1354 Wheeze (at 23–29 mo): Maternal report wheezing symptom in the last 12 months (ISAAC questionnaire), n = 1354
Anderson, 2015 [55]	Toronto, Ontario, Canada (43°40’N)	Maternal vitamin D supplementation	questionnaire	(1) During pregnancy (retrospective review at 0–5 ages) (2) 0–5 ages	Yes/no	Asthma (2.3 years after baseline): parent-reported diagnosed, *n* = 2478 Wheezing(2.3 years after baseline): parent-reported (ISAAC questionnaire), *n* = 484
Back, 2009 [56]	Umeå, Sweden (63°49’N)	Infantile intake (total)	FFQ	5, 7 and 10 months during infancy	Low (≤13.0 μg/d)/ high (> 13.0 μg/d) dose	Asthma (6y): Unclear definition (ISAAC questionnaire), *n* = 123
Hypponen, 2004 [57]	Oulu (65° 0′46”N), Lapland (61°2′45”N), Finland	Infantile intake (supplement)	Maternal report	Infancy	None or irregularly/regularly	Asthma (31y): Self-report of asthma with wheezing or by current use of asthma medication, *n* = 6722

BMI: body mass index; ELISA: enzyme-linked immunosorbent assay; FFQ: food frequency questionnaire; HPLC: high performance liquid chromatography; ISAAC: The International Study of Asthma and Allergies in Children; LC-MS/MS: liquid chromatography-tandem mass spectrometry, IQR: interquartile range, SD: standard deviation

*Results from the same study: pooled estimates of asthma and wheeze at >3y using results from Pike et al.; wheeze at <3y from De Jongh et al.

**Results from the same study: pooled estimates of asthma using results from Allan et al.; wheeze from Devereux et al.

# Not included in the meta-analysis of blood vitamin D levels as the continuous variables did not provide sufficient data to calculate estimates

### Early life vitamin D status and asthma

Two RCTs [27, 28] reported non-significant trends of vitamin D supplementation during pregnancy on preventing the development of offspring asthma (OR, 0.82; 95% CI 0.50–1.36 and HR, 0.8; 95% CI, 0.6–1.0, respectively). As the intervention and the outcome definition were different, we did not pool the effect size. Apart from having insufficient information to assess allocation concealment, risk of bias was low in both RCTs (Additional file 1: Figure S1).

**Fig. 2:**
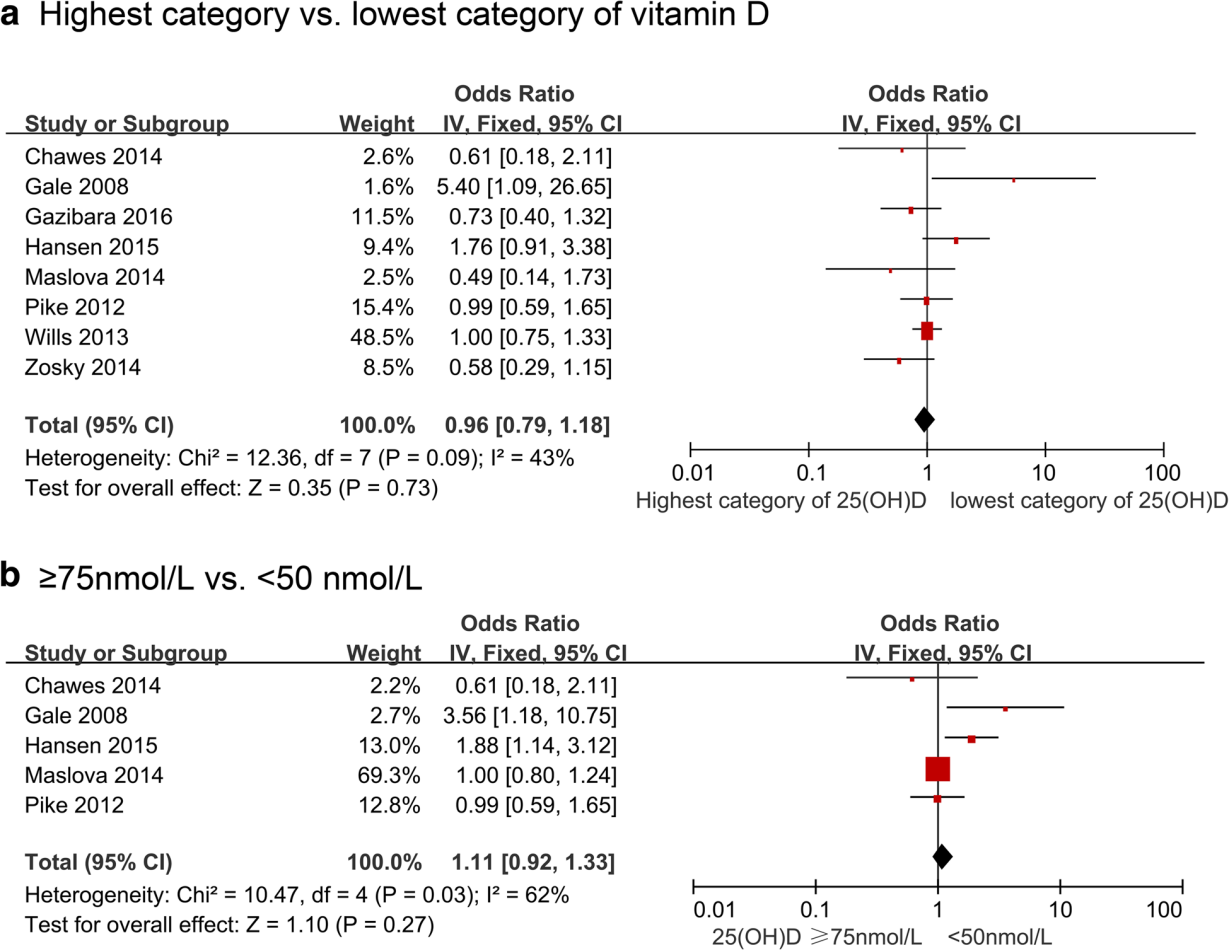
Study-specific and summary effects of pooled risk estimates of blood vitamin D levels and asthma assessed > 5 years in cohort studies. **a** Highest category vs. lowest category of vitamin D; **b** ≥75 nmol/L vs. < 50 nmol/L

Figure 2 and Table 3 show the results from cohort studies as well as overall and subgroup summary ORs for the relationship between blood vitamin D and the risk of asthma assessed at > 5 years of age. Fix-effects ORs were not significant for the highest vs. the lowest categories of blood vitamin D concentrations (8 cohort studies, 28,436 participants, Fig. 2a) and for the sufficient vs. deficient groups **(**5 cohort studies, 23,339 participants, Fig. 2b), with moderate heterogeneity. The pooled estimate did not change materially (OR 0.91, 95% CI 0.73, 1.12, I^2^ = 32%, for highest vs. lowest; OR 1.02, 95% CI 0.84, 1.24, I^2^ = 47% for sufficient vs. deficient groups) after excluding one study assessing asthma in adulthood. Little heterogeneity was observed across subgroups, in studies measuring cord blood vitamin D level, adjusted for family history of allergic disease, or seasonality, those with exposure measured by liquid chromatography-tandem mass spectrometry (LC-MS/MS), and studies with moderate risk of bias (Table 3).

**Table 3:** Summary ORs of asthma and early life blood vitamin D levels (highest versus lowest category) in cohort studies, according to selected subgroups

Age at outcome assessment >5y					Age at outcome assessment ≤5y			
Subgroup	Studies (Ref. no.)	No. of studies	OR (95% CI)	I^2^	Studies (Ref. no.)	No. of studies	OR (95% CI)	I^2^
Window of exposure assessment								
During pregnancy (mother)	[20, 22, 31–33, 36, 37]	7	0.98 [0.80, 1.20]	49%	[30, 32, 34, 35, 45]	5	0.79 [0.63, 0.99]	0%
Cord blood	[37, 41]	2	1.03 [0.60, 1.79]	0%	[23]	1	2.00 [0.48, 8.31]	–
Period of outcome assessment								
At an end point (e.g. at age 1y)	[20, 22, 31–33, 41]	6	0.94 [0.75, 1.17]	39%	[30, 32, 34]	3	0.81 [0.64, 1.02]	20%
Up to an end point (e.g. by age 1y)	[32, 36, 37]	3	1.12 [0.77, 1.64]	49%	[23, 35, 45]	3	0.83 [0.40, 1.75]	17%
Latitude								
Tropics or subtropics (more intense UVR) (≤40°N/S)	[31]	1	0.58 [0.29, 1.15]	–	[23, 30, 35, 45]	4	0.87 [0.61, 1.24]	0%
Temperate (40–66.5°N/S)	[20, 22, 32, 33, 36, 37, 41]	7	1.01 [0.82, 1.25]	40%	[32, 34]	2	0.77 [0.57, 1.03]	54%
Region								
Europe	[20, 22, 32, 33, 36, 37, 41]	7	1.01 [0.82, 1.25]	40%	[30, 32, 34]	3	0.81 [0.64, 1.02]	20%
USA/Canada					[23]	1	2.00 [0.48, 8.31]	–
Australia	[31]	1	0.58 [0.29, 1.15]	–	[45]	1	0.74 [0.26, 2.14]	–
Asia					[35]	1	0.40 [0.09, 1.80]	–
Adjusted for family history of atopy								
No	[20, 33, 36]	3	1.14 [0.88, 1.48]	67%	[23, 34, 35]	3	0.70 [0.50, 0.96]	25%
Yes	[22, 31, 32, 37, 41]	5	0.75 [0.55, 1.03]	0%	[30, 32, 45]	3	0.93 [0.68, 1.28]	0%
Adjusted for seasonality								
No	[20, 22, 31]	3	0.92 [0.62, 1.37]	69%	[30, 45]	2	0.87 [0.60, 1.26]	0%
Yes	[32, 33, 36, 37, 41]	5	0.98 [0.78, 1.24]	30%	[23, 32, 34, 35]	4	0.78 [0.59, 1.03]	35%
Adjusted for smoking statues								
No	[20, 22, 33]	3	1.04 [0.81, 1.33]	52%	[35, 45]	2	0.60 [0.25, 1.44]	0%
Yes	[31, 32, 36, 37, 41]	5	0.84 [0.60, 1.18]	44%	[23, 30, 32, 34]	4	0.83 [0.66, 1.04]	26%
Outcome assessment								
Maternal report of doctor diagnosed or medication only	[22, 36, 37]	3	1.04 [0.75, 1.45]	49%	[23, 30, 45]	3	0.92 [0.64, 1.32]	0%
Maternal report of doctor diagnosed plus medication and/or wheeze symptom	[31–33]	3	0.90 [0.69, 1.16]	33%	[32, 34, 35]	3	0.75 [0.57, 1.00]	31%
Doctor diagnosed or medical review	[41]	1	0.61 [0.18, 2.13]	–				
Other definition	[20]	1	5.40 [1.09, 26.65]	–				
Blood 25(OH)D measurement								
LC-MS/MS (gold standard)	[32, 33, 36, 37, 41]	5	0.98 [0.78, 1.24]	30%	[23, 30, 32, 34, 45]	5	0.82 [0.66, 1.03]	2%
Other method	[20, 22, 31]	3	0.92 [0.62, 1.37]	69%	[35]	1	0.40 [0.09, 1.80]	–
Risk of bias								
Moderate	[22, 30, 32, 33, 36, 37, 41]	6	0.98 [0.80, 1.21]	13%	[23, 30, 32, 34, 45]	5	0.82 [0.66, 1.03]	2%
High	[20, 31]	2	1.57 [0.18, 13.80]	84%	[35]	1	0.40 [0.09, 1.80]	–
Overall	[20, 22, 31–33, 36, 37, 41]	8	0.96 [0.79, 1.18]	43%	[23, 30, 32, 34, 35, 45]	6	0.81 [0.65, 1.01]	0%

ISAAC: The International Study of Asthma and Allergies in Children; LC-MS/MS: liquid chromatography-tandem mass spectrometry; UVR: ultraviolet radiation

**Fig. 3:**
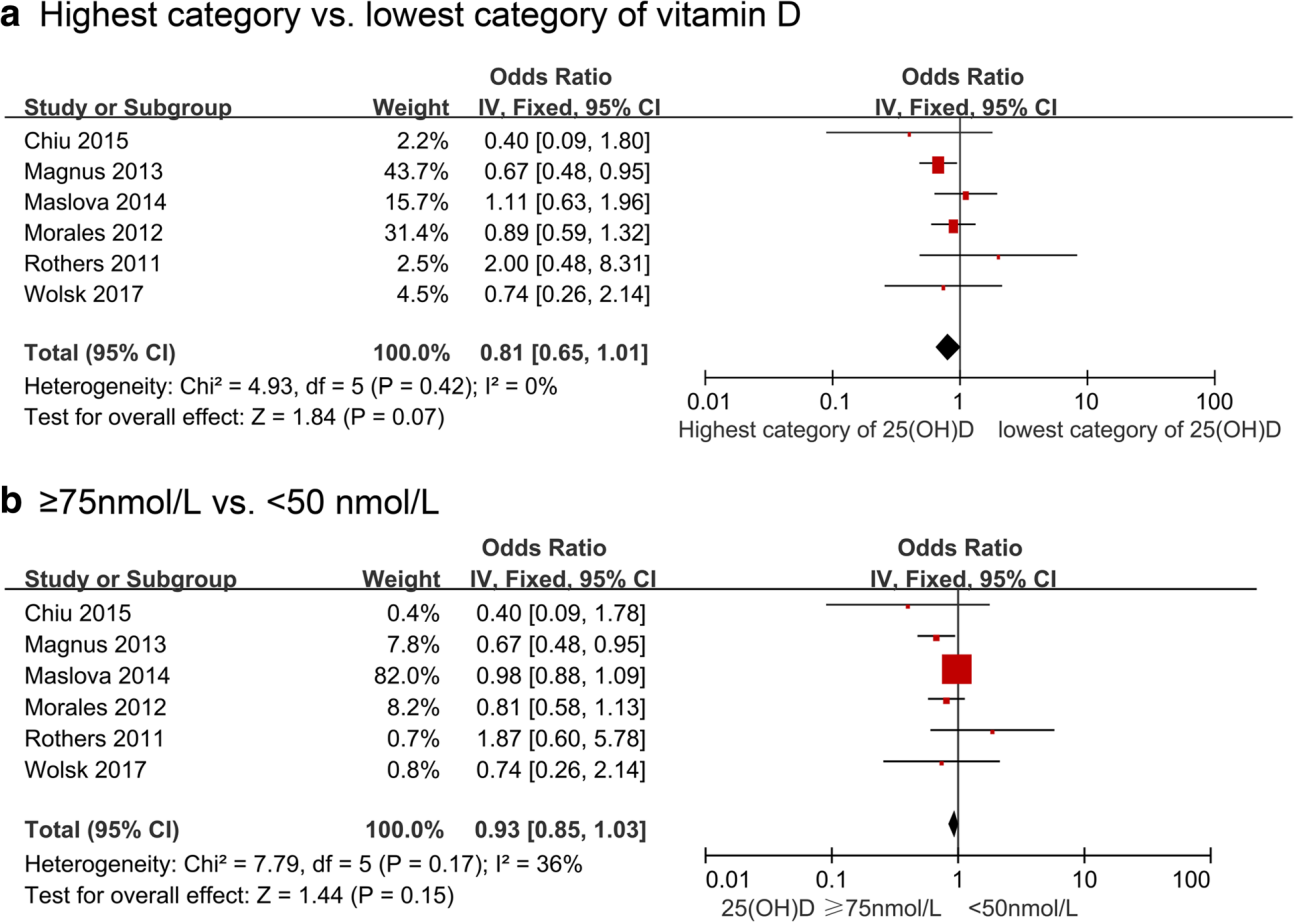
Study-specific and summary effects of pooled risk estimates of blood vitamin D levels and asthma assessed **≤**5 years in cohort studies. **a** Highest category vs. lowest category of vitamin D; **b** ≥75 nmol/L vs. < 50 nmol/L

Figure 3 and Table 3 show the results from cohort studies and the overall and subgroup summary ORs for the relationship between blood vitamin D and the risk of asthma assessed at ≤5 years of age. The fixed-effects ORs were 0.81 (95% CI 0.65, 1.01, I^2^ = 0%, 6 cohort studies, 27,776 participants, Fig. 3a) for the highest vs. lowest categories of blood vitamin D concentrations and 0.93 for the sufficient vs. deficient groups **(**95% CI 0.85, 1.03, I^2^ = 36%, 6 cohort studies**,** 27,776 participants, Fig. 3b). Although higher maternal blood vitamin D level during pregnancy was significantly associated with lower risk of asthma assessed at ≤5 years, we did not observe any significant associations in the stratified analyses for studies that had more robust methodology (adjusting for further confounders, e.g. family history of atopy, seasonality, smoking status; blood calcifediol measured by the gold standard LC-MS/MS; studies with lower risk of bias) (Table 3). Six eligible cohort studies [38, 39, 43, 44, 46, 47] analyzed blood vitamin D as a continuous variable and we found no association with childhood asthma.

There was no association between vitamin D intake and asthma at ≤5 years (6 cohorts, 35,257 participants) or > 5 years (3 cohorts, 32,023 participants) (Table 4). A significant inverse association between vitamin D intake and asthma at ≤5 years of age emerged when we restricted our analysis to studies that did not adjust for seasonality or had high risk of bias, with low heterogeneity within these subgroups; and between vitamin D intake and asthma at > 5 years of age when we restricted to studies that adjusted for family history of atopy, seasonality or smoking status, or those that assessed outcome in childhood (OR 0.73, 95% CI 0.56, 0.94, I^2^ = 0%, 25,301 participants from 2 studies, for highest vs. lowest) (Table 4). The number needed to treat to prevent one case of asthma was 39 (range 25–93) via maternal vitamin D supplementation (Additional file 1: Table S2).

**Table 4:** Summary ORs of asthma and early life vitamin D intake (highest versus lowest category) in cohort studies, according to selected subgroups

Age at outcome assessment >5y					Age at outcome assessment ≤5y			
Subgroup	Studies (Ref. no.)	No. of studies	OR (95% CI)	I^2^	Studies (Ref. no.)	No. of studies	OR (95% CI)	I^2^
Window of exposure assessment								
During pregnancy (mother)	[50, 57]	2	1.21 [0.90, 1.62]	64%	[49, 53, 55, 56]	4	0.89 [0.77, 1.04]	68%
During infancy	[52]	1	0.74 [0.56, 0.96]	–	[52, 54]	2	0.95 [0.85, 1.06]	27%
Period of outcome assessment								
At an end point (e.g. at age 1y)	[50, 52, 57]	3	0.90 [0.56, 1.45]	77%	[54]	1	0.60 [0.27, 1.31]	–
Up to an end point (e.g. by age 1y)	[50]	1	0.60 [0.32, 1.11]	–	[49, 52, 53, 55, 56]	5	0.94 [0.86, 1.02]	60%
Latitude								
Tropics or subtropics (more intense UVR) (≤40°N/S)	–	–	–	–	[54]	1	0.60 [0.27, 1.31]	–
Temperate (40–66.5°N/S)	[50, 52, 57]	3	0.90 [0.56, 1.45]	77%	[49, 52, 53, 55, 56]	5	0.94 [0.86, 1.02]	60%
Region								
Europe	[50, 52, 57]	3	0.90 [0.56, 1.45]	77%	[49, 52, 53, 56]	4	0.96 [0.88, 1.05]	47%
USA/Canada					[55]	1	0.65 [0.46, 0.93]	–
Asia					[54]	1	0.60 [0.27, 1.31]	–
Adjusted for family history of atopy								
No	[57]	1	1.33 [0.97, 1.82]	–	[55]	1	0.65 [0.46, 0.93]	–
Yes	[50, 52]	2	0.73 [0.56, 0.94]	0%	[49, 52–54, 56]	5	0.95 [0.87, 1.04]	43%
Adjusted for seasonality								
No	[57]	1	1.33 [0.97, 1.82]	–	[53–56]	4	0.64 [0.48, 0.86]	29%
Yes	[50, 52]	2	0.73 [0.56, 0.94]	0%	[49, 52]	2	0.97 [0.88, 1.06]	0%
Adjusted for smoking statues								
No	[57]	1	1.33 [0.97, 1.82]	–	[56]	1	3.16 [0.61, 16.32]	–
Yes	[50, 52]	2	0.73 [0.56, 0.94]	0%	[49, 52–55]	5	0.93 [0.85, 1.01]	56%
Outcome assessment								
Maternal report of doctor diagnosed or medication only	[50]	1	0.60 [0.32, 1.11]	–	[52, 53]	2	0.94 [0.85, 1.05]	71%
Maternal report of doctor diagnosed plus medication and/or wheeze symptom	[50, 52, 57]	3	0.90 [0.56, 1.45]	77%	[49, 55]	2	0.82 [0.55, 1.24]	77%
Doctor diagnosed or medical review	–	–	–	–	[54]	1	0.60 [0.27, 1.31]	–
Based on ISAAC questionnaire	–	–	–	–	[56]	1	3.16 [0.61, 16.32]	–
Risk of bias								
Moderate	[52, 57]	2	0.98 [0.55, 1.75]	87%	[49, 52, 54, 56]	4	0.96 [0.88, 1.06]	14%
High	[50]	1	0.63 [0.28, 1.44]	–	[53, 55]	2	0.61 [0.45, 0.84]	0%
Overall	[50, 52, 57]	3	0.90 [0.56, 1.45]	77%	[49, 52–56]	6	0.93 [0.85, 1.02]	56%

ISAAC: The International Study of Asthma and Allergies in Children; LC-MS/MS: liquid chromatography-tandem mass spectrometry; UVR: ultraviolet radiation

Studies that measured blood vitamin D concentrations generally had a low risk of bias except for attrition bias due to incomplete outcome data, confounding bias, and detection bias in the assessment of prognostic factors (Additional file 1: Figures S2 and S3). Several studies that assessed vitamin D intake had a high risk of detection bias due to recall of vitamin D intake, confounding bias, and attrition bias due to incomplete outcome data. (Additional file 1: Figures S4 and S5). No evidence of publication bias was found from the funnel plots or Egger’s tests for blood (*P* = 0.963 for asthma > 5 years and *P* = 0.655 for asthma ≤5 years) and intake (*P* = 0.913 for asthma > 5 years and *P* = 0.410 for asthma ≤5 years) (Additional file 1: Table S3).

### Early life vitamin D status and wheeze

All three RCTs [26–28] used wheeze as primary outcome (one combined with asthma [28]). Two RCTs found no definitive effect of vitamin D supplementation during pregnancy on wheeze among offspring by 3 years of age (OR 0.86; 95% CI 0.49, 1.50; HR 0.76; 95% CI 0.52, 1.12) and one found a borderline statistical significance association (HR 0.8; 95% CI 0.6, 1.0). We did not perform pooled analysis for wheeze because of the major differences in intervention. Risks of performance and attrition bias were considered to be high due to missing outcome data and the lack of blinding in the trial conducted by Goldring et al. [26] (Additional file 1: Figure S1).

**Fig. 4:**
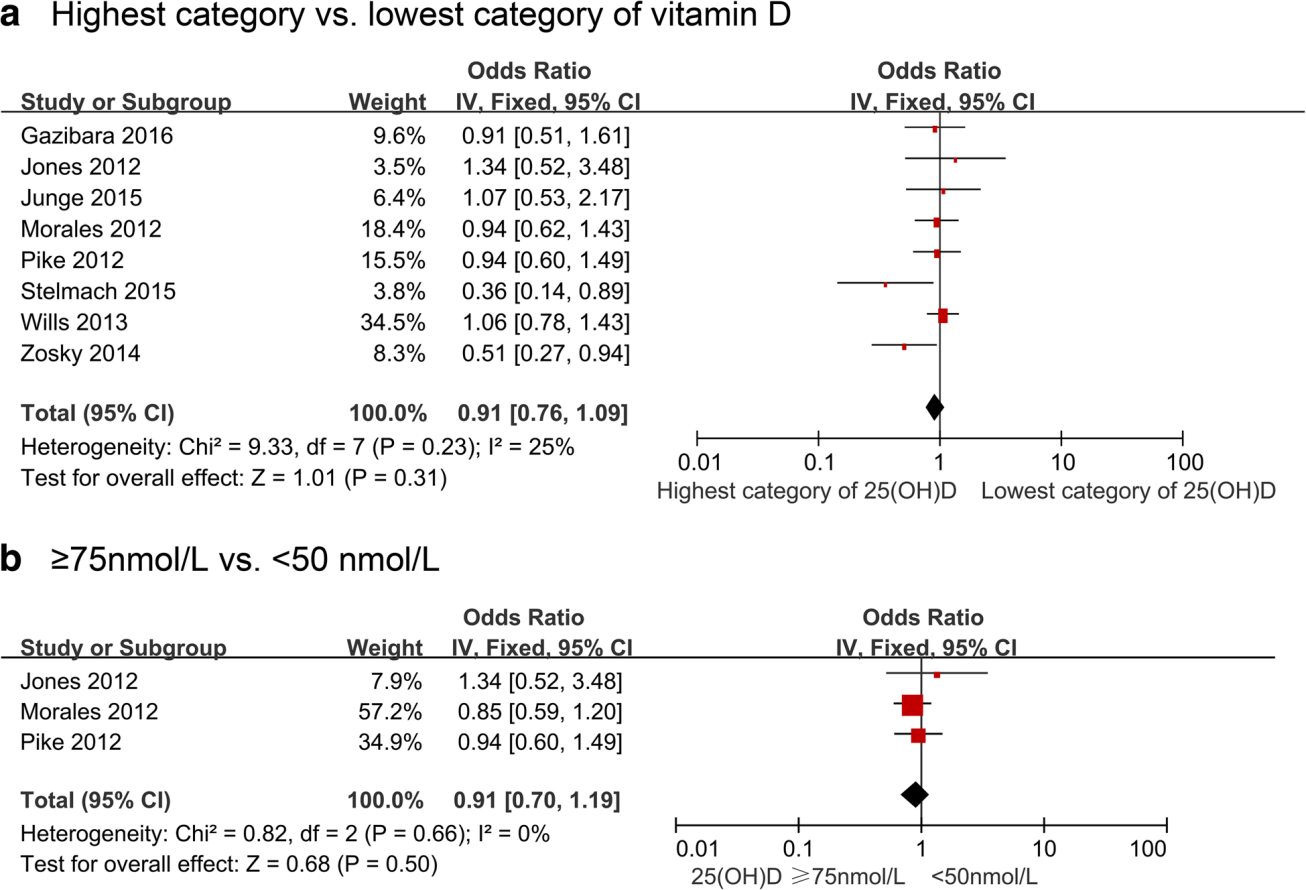
Study-specific and summary effects of pooled risk estimates of blood vitamin D levels and wheeze in cohort studies. **a** Highest category vs. lowest category of vitamin D; **b** ≥75 nmol/L vs. < 50 nmol/L

We did not observe significant association between maternal vitamin D concentrations during pregnancy and offspring wheeze in cohort studies (8123 participants from 8 studies, OR 0.91, 95% CI 0.76, 1.09 for highest vs. lowest levels, I^2^ = 25%; 2324 participants from 3 studies, OR 0.91, 95% CI 0.70, 1.19 for ≥75 nmol/L vs. < 50 nmol/L, I^2^ = 0%) (Fig. 4). Similar results were observed in stratified analyses (Table 5). Within-group inconsistency disappeared or was attenuated in studies that measured vitamin D intake during pregnancy or adjusted for family history of atopy or seasonality or blood calcifediol measured by LC-MS/MS or studies with moderate risk of bias. The overall fixed-effects OR of wheeze relating to maternal vitamin D intake was 0.66 (95% CI 0.53, 0.82; 5238 participants from 6 studies, I^2^ = 52%, Table 5). The association remained statistically significant when restricting to studies that: assessed outcome at or before 3 years of age and  after 3 years of age, were conducted in a temperate area, adjusted for family history of atopy or seasonality, and those that had lower risk of bias. The Baiz paper [38] did not find an association in early transient, late onset or persistent wheeze, while Camargo et al. [39] reported a significant protective effect by 5 years of age (OR 0.95, 95% CI 0.91, 0.99), but this has not been consistent in earlier time windows reported in the studies by Camargo et al. [39] and Visness et al. [44]

**Table 5:** Summary ORs of wheeze and vitamin D status (highest versus lowest category) in cohort studies, according to selected subgroups

Early life blood vitamin D levels					Early life vitamin D intake			
Subgroup	Studies (Ref. no.)	No. of studies	OR (95% CI)	I^2^	Studies (Ref. no.)	No. of studies	OR (95% CI)	I^2^
Window of exposure assessment								
During pregnancy (mother)	[22, 30, 31, 33, 37]	5	0.93 [0.76, 1.12]	9%	[21, 48, 49, 51, 54, 55]	6	0.66 [0.53, 0.82]	52%
Cord blood	[21, 40, 42]	3	0.83 [0.51, 1.35]	58%	–	–	–	–
During infancy (infant)	[42]	1	1.88 [0.89, 3.98]					
Age at outcome assessment								
≤3y	[21, 29, 30, 37, 40, 42]	6	0.93 [0.77, 1.12]	26%	[21, 48, 51, 54]	4	0.68 [0.53, 0.88]	63%
> 3y	[22, 30, 31, 33, 37]	5	1.09 [0.89, 1.33]	11%	[49, 55]	2	0.57 [0.35, 0.93]	48%
Latitude								
Tropics or subtropics (more intense UVR) (≤40°N/S)	[21, 30, 31]	3	0.83 [0.60, 1.14]	47%	[21, 48, 54]	3	0.81 [0.61, 1.07]	7%
Temperate (40–66.5°N/S)	[22, 33, 37, 40, 42]	5	0.95 [0.77, 1.18]	21%	[49, 51, 55]	3	0.47 [0.33, 0.68]	35%
Adjusted for family history of atopy								
No	[40]	1	0.36 [0.14, 0.89]	–	[21, 55]	2	0.84 [0.47, 1.49]	46%
Yes	[21, 22, 30, 31, 33, 37, 42]	7	0.95 [0.79, 1.14]	0%	[48, 49, 51, 54]	4	0.63 [0.49, 0.80]	61%
Adjusted for seasonality								
No	[22, 30, 31, 40]	4	0.78 [0.60, 1.01]	51%	[21, 48, 54, 55]	4	0.79 [0.61, 1.03]	0%
Yes	[21, 33, 37, 42]	4	1.05 [0.82, 1.33]	0%	[49, 51]	2	0.37 [0.24, 0.58]	0%
Blood 25(OH)D measurement								
LC-MS/MS (gold standard)	[21, 30, 33, 37, 40, 42]	6	0.97 [0.79, 1.19]	10%	–	–	–	–
Other methods	[22, 31]	2	0.76 [0.53, 1.10]	60%	–	–	–	–
Risk of bias								
Moderate	[22, 30, 33, 37]	4	0.99 [0.81, 1.21]	0%	[48, 49, 51, 54]	4	0.63 [0.49, 0.80]	61%
High	[21, 31, 40, 42]	4	0.69 [0.47, 1.01]	52%	[21, 55]	2	0.84 [0.47, 1.49]	46%
Overall	[21, 22, 30, 31, 33, 37, 40, 42]	8	0.91 [0.76, 1.09]	25%	[21, 48, 49, 51, 54, 55]	6	0.66 [0.53, 0.82]	52%

LC-MS/MS: liquid chromatography-tandem mass spectrometry

The 12 cohort studies that investigated blood vitamin D levels and wheeze were evaluated to have low risk of bias, in terms of participant selection, exposure assessment, and window of outcome assessment and co-intervention. Some studies had high or unclear risk of confounding bias, detection bias and attrition bias (Additional file 1: Figure S6, a and b). Overall, the studies on vitamin D intake had higher risk of bias, due to their inadequacy in exposure assessment, assessment and adjustment for prognostic factors, and outcome follow-up (Additional file 1: Figure S7, a and b). There was no evidence of publication bias from the funnel plots or Egger’s tests for blood (*P* = 0.772) and intake (*P* = 0.954) (Additional file 1: Table S3).

## Discussion

This meta-analysis of cohort studies found no statistically significant association between vitamin D levels in maternal or cord blood or intake in early life and asthma either at > 5 or ≤ 5 years of age, with no evidence of publication bias. Of the cohort studies included, only two studies assessed asthma in adulthood [36, 57]. The exclusion of these two studies did not change the pooled estimate for blood vitamin D levels, while a significant inverse association between early life vitamin intake and childhood asthma at > 5 years emerged. We found no association between early life vitamin D level and risk of wheeze in later life. On the other hand, findings from cohort studies seemed to suggest a lower risk of wheeze associated with maternal vitamin D intake during pregnancy.

Variations in the intervention and outcome definition in three identified RCTs made it difficult to pool the effect size for wheeze or asthma associated with vitamin D supplementation during pregnancy. Two more recent trials [27, 28] with lower risk of bias also found non-statistically significant benefit for persistent/recurrent wheeze or asthma in the first 3 years of life. The results of RCTs were in line with the results of our meta-analysis of cohort studies, where we found non-significant trends of vitamin D supplementation for preventing offspring asthma at ≤5 years. However, our findings from cohort studies also suggest that early-life vitamin D intake may have an effect on childhood asthma at age > 5 years and childhood wheeze. Longer follow-up time for assessing the effect of vitamin D intake against asthma is required in future RCTs to confirm these results [58].

There are two opposing views regarding the relationship between vitamin D exposure and asthma. One school of thought, based on growing epidemiological evidence, hypothesized a link between vitamin D deficiency in early life and development of asthma and other allergic diseases [59], thought to have begun as sunlight exposure decreased with industrialization and urbanization. Others argued, however, that the current asthma and allergic disease pandemic might have been the consequence of widespread vitamin D supplementation in food, backed by some historical evidence [60]. Coincidentally, both low and high levels of vitamin D were reported to be related to asthma, in a U-shaped manner [32]. Another study by Rothers et al. also found that both low (< 50 nmol/L) and high (≥100 nmol/L) levels of cord blood calcifediol were associated with increased IgE, which was related to subsequent risk of wheeze/asthma [23]. In the present meta-analysis, the inconsistent results from studies assessing vitamin D either in blood or intake for childhood asthma/wheeze risk may be explained by the previously reported non-linear association between blood vitamin D_3_ and childhood asthma, which tends to be non-statistically significant by comparing the highest with lowest vitamin D levels [32]. However, the dichotomized exposure in the present study limited the exploration of non-linear association.

The sources of heterogeneity, as indicated by the results from stratified analyses were: window of exposure assessment, whether family history of atopy or seasonality have been adjusted for, definition of outcome, risk of bias, and the method of assessing blood calcifediol. As suggested by Autier et al. [61], vitamin D level could be a biomarker and proxy for overall health and wellbeing of mothers and infants. However, as most of the cohort studies included did not adjust for health status of the participants, we were unable to exclude such potential confounding. In most of the studies included, tobacco smoking was adjusted for in the analysis, but other important confounders including family history of atopic diseases, seasonal variations in blood vitamin D levels, sedentary lifestyle and obesity [62–64] were less commonly considered. Nevertheless, results from our sensitivity analysis on studies reporting blood vitamin D level, which included only studies that adjusted for family history and seasonal variations, showed no material difference in the summary estimates compared with our main findings. Three of the cohort studies [35, 42, 47] measured serum calcifediol in both prenatal and postnatal periods, but these studies analyzed the associations between vitamin D measured at different time points with outcome separately and failed to explore the effect of persistent vitamin D deficiency on asthma. While the half-life of serum calcifediol is only about two weeks [65], it is possible that such misclassification in vitamin D status measured at only one time point could have biased the findings towards the null. A recent study conducted by Hollams et al. used mixed-effects logistic regression and found no longitudinal association between calcifediol concentrations as a continuous variable and asthma from 3 to 10 years of age [46].

On the issue of case definition, ascertaining childhood asthma in epidemiological studies has been challenging given the lack of definitive diagnostic criteria [66, 67]. Most studies defined asthma by either maternal report of doctor-diagnosed asthma, asthma medications use, or a combination of doctor-diagnosed asthma and either asthma medication or wheezing symptoms within the past year. Although not without caveats, such as under-reporting both diagnosed and undiagnosed conditions [68] and variable agreement with clinical records [67], these definitions have been widely used in research [69]. In two studies asthma definition was not specified, adding further variability to outcome assessment and increased heterogeneity in our pooled analyses.

In general, studies investigating blood vitamin D levels had relatively low risk of bias in most domains except for confounding, detection, and attrition biases, whereas studies that used food frequency questionnaire to quantify vitamin D intake were at risk of misclassification bias as pregnant women often had to recall their dietary history from a few months previous.. Apart from detection bias, most vitamin D intake studies were also at risk of attrition bias for their inadequate follow-up duration or unknown difference between missing and available outcome data, as well as confounding bias in some studies which did not include major confounders. In addition to variation over time, different methods of blood vitamin D level quantification employed in different studies might also introduce inter-study variability. In the stratified analysis, we compared the risk estimates derived from studies that used the gold standard LC-MS/MS with those using other methods and found no material difference between the pooled estimates. Studies that investigated antenatal vitamin D intake and asthma at > 5 years of age tended to be small and their inclusion in the meta-analysis may explain the weak evidence observed.

Compared to previous related systematic reviews [11–13, 70], we have identified more cohort studies and included two recently published RCTs in the analysis. Only six cohort studies [20, 22, 23, 30, 33, 39] measuring maternal serum calcifediol during pregnancy or from cord blood and asthma risk were identified by Cassim et al. [70], but we have included 13 more studies [31, 32, 34–38, 41, 43–47], plus an additional 13 cohort studies [21, 22, 29–31, 33, 37–40, 42, 44, 46] to explore the association between calcifediol level in maternal blood or cord blood and wheeze risk. As a consequence of our more comprehensive approach in including various exposure metrics (intake and blood level, both maternal and in infant) in the search strategy and a critical risk of bias assessment using the risk of bias tool recommended by the Cochrane Collaboration [17, 18], we were able to demonstrate the existence of heterogeneity between studies in the findings, which lead us to a more conservative conclusion. In addition, we further studied the association between antenatal or early postnatal vitamin D level and asthma and wheeze using commonly accepted clinical cut-off values. We found no difference of risk of asthma or wheeze between sufficient and deficient vitamin D level groups, implying the optimal threshold for bone health may not necessarily be applicable for the respiratory and immune systems.

One of the limitations of this work is that we were unable to retrieve sufficient information for calculating pooled estimates from six eligible studies that analyzed vitamin D level as a continuous variable. We were able to identify one study that has examined infant blood vitamin D levels and their relationship to wheeze but none for asthma. Studies investigating the association between infant vitamin D levels and risk of childhood asthma should be encouraged, with designs that minimize detection, confounding, and follow-up biases.

## Conclusions

The pooled estimates from cohort studies show no association between antenatal blood vitamin D level and asthma/wheeze in later life. On the other hand, although the interventional studies found a non-statistically significant benefit for asthma/wheeze in first 3 years of life, the pooled estimates from cohort studies suggest that early life vitamin D intake may have an effect on childhood asthma > 5 years or childhood wheeze. Should that be the case, this may point to a cost-effective intervention for childhood asthma. Further trials with enough power and longer follow-up time should be conducted to confirm the results.

## Additional file

Additional file 1: Supplementary Materials. (DOCX 290 kb).

## Abbreviations

BMI: body mass index
CIs: confidence intervals
ELISA: enzyme-linked immunosorbent assay
FFQ: food frequency questionnaire
HPLC: high performance liquid chromatography
HR: hazard ratio
IOM: Institute of Medicine
ISAAC: The International Study of Asthma and Allergies in Children
LC-MS/MS: liquid chromatography-tandem mass spectrometry
MOOSE: Meta-analysis of Observational Studies in Epidemiology
OR: odds ratio
PRISMA: Preferred Reporting Items for Systematic Reviews and Meta-Analyses
RCT: randomized controlled trial
RR: relative risk
UVR: ultraviolet radiation
