# Supplementary material for: Early life vitamin D status and asthma and wheeze: a systematic review and meta-analysis
Source: BMC Pulm Med. 2018 Jul 20;18:120. doi: 10.1186/s12890-018-0679-4 (PMC6053833; doi:10.1186/s12890-018-0679-4)
Supplement: Supplementary file 1 — Supplementary Materials. (DOCX 290 kb). [file 12890_2018_679_MOESM1_ESM.docx]

**Early life vitamin D status and asthma and wheeze: a systematic review and meta-analysis**

Songying Shen ^1^, MMs; Wanqing Xiao ^1^, MMs; Jinhua Lu ^1^, MMs; Mingyang Yuan^1^, MMs; Jianrong He ^1^, MMs; Huimin Xia^2^, MMs; Xiu Qiu ^1*^, PHD; Kar Keung Cheng ^3^, PHD; Kin Bong Hubert Lam^3,4^,PHD

^1^ Division of Birth Cohort Study, Guangzhou Women and Children’s Medical Center, Guangzhou Medical University, Guangzhou, China;

^2^ Department of Pediatric Surgery, Guangzhou Women and Children's Medical Center, Guangzhou Medical University, Guangzhou, China;

^3^ Unit of Public Health, Epidemiology and Biostatistics, University of Birmingham, UK;

^4^ Nuffield Department of Population Health, University of Oxford, UK.

*Corresponding author: Xiu Qiu, Division of Birth Cohort Study, Guangzhou Women and Children’s Medical Center, Guangzhou Medical University, 9 Junsui Road, Zhujiang Newtown, Tianhe District, Guangzhou 510623, China. Email: [qxiu0161@163.com](mailto:qxiu0161@163.com). Tel/Fax: +86-20-38367162 (O)

**Supplementary Materials**

**Methods**

Supplement 1 Completed PRISMA checklist and MOOSE checklist.

Supplement 2 Search strategy and results in Pubmed and Embase databases.

Supplement 3 Form for guidance of title and abstract screening process.

Supplement 4 Form for guidance of full text assessment process.

Supplement 5 Details on methods of data extraction and risk of bias assessment.

**Tables**

Table S1 Characteristics of studies excluded by full-text screening.

Table S2 The Number Needed to Treat estimate for preventing asthma by increasing from low to high levels of vitamin D or via supplementation.

Table S3 Publication bias of the included studies by study design.

**Figures**

Figure S1 Risk of bias for randomized controlled trials on maternal vitamin D supplementation and offspring asthma/wheeze.

Figure S2 Risk of bias for cohort studies on early life blood vitamin D levels and asthma assessed >5 years..

Figure S3 Risk of bias for cohort studies on early life blood vitamin D levels and asthma assessed ≤5 years..

Figure S4 Risk of bias for cohort studies on early life vitamin D intake and asthma assessed >5 years.

Figure S5 Risk of bias for cohort studies on early life vitamin D intake and asthma assessed ≤5 years.

Figure S6 Risk of bias for cohort studies on early life blood vitamin D level and wheeze.

Figure S7 Risk of bias of cohort studies on early life vitamin D intake and wheeze.

**Supplement 1. Completed PRISMA Checklist**

**PRISMA 2009 Checklist**

| **Section/topic** | **#** | **Checklist item** | **Reported on page #** |
| --- | --- | --- | --- |
| **TITLE** | | |  |
| Title | 1 | Identify the report as a systematic review, meta-analysis, or both. | Page 1 |
| **ABSTRACT** | | |  |
| Structured summary | 2 | Provide a structured summary including, as applicable: background; objectives; data sources; study eligibility criteria, participants, and interventions; study appraisal and synthesis methods; results; limitations; conclusions and implications of key findings; systematic review registration number. | Page 3-4 |
| **INTRODUCTION** | | |  |
| Rationale | 3 | Describe the rationale for the review in the context of what is already known. | Page 5, paragraph 1-2 |
| Objectives | 4 | Provide an explicit statement of questions being addressed with reference to participants, interventions, comparisons, outcomes, and study design (PICOS). | Page 5, paragraph 3 |
| **METHODS** | | |  |
| Protocol and registration | 5 | Indicate if a review protocol exists, if and where it can be accessed (e.g., Web address), and, if available, provide registration information including registration number. | Page 6, paragraph 1 |
| Eligibility criteria | 6 | Specify study characteristics (e.g., PICOS, length of follow-up) and report characteristics (e.g., years considered, language, publication status) used as criteria for eligibility, giving rationale. | Page 6, paragraph 3 and supplement 3 |
| Information sources | 7 | Describe all information sources (e.g., databases with dates of coverage, contact with study authors to identify additional studies) in the search and date last searched. | Page 6, paragraph 2 |
| Search | 8 | Present full electronic search strategy for at least one database, including any limits used, such that it could be repeated. | Supplement 2 |
| Study selection | 9 | State the process for selecting studies (i.e., screening, eligibility, included in systematic review, and, if applicable, included in the meta-analysis). | Page 6, Paragraph 3; Page 7 paragraph 1 and supplement 3 and supplement 4 |
| Data collection process | 10 | Describe method of data extraction from reports (e.g., piloted forms, independently, in duplicate) and any processes for obtaining and confirming data from investigators. | Page 7, paragraph 2 and supplement 4 |
| Data items | 11 | List and define all variables for which data were sought (e.g., PICOS, funding sources) and any assumptions and simplifications made. | Page 7, paragraph 2 and supplement 5 |
| Risk of bias in individual studies | 12 | Describe methods used for assessing risk of bias of individual studies (including specification of whether this was done at the study or outcome level), and how this information is to be used in any data synthesis. | Page 7, paragraph 2 and supplement 5 |
| Summary measures | 13 | State the principal summary measures (e.g., risk ratio, difference in means). | Page 8, paragraph 2 and supplement 5 |
| Synthesis of results | 14 | Describe the methods of handling data and combining results of studies, if done, including measures of consistency (e.g., I2) for each meta-analysis. | Page 8, paragraph 3-4 and page 9 paragraph 1 |

**PRISMA 2009 Checklist**

| **Section/topic** | **#** | **Checklist item** | **Reported on page #** |  |  |
| --- | --- | --- | --- | --- | --- |
| Risk of bias across studies | 15 | Specify any assessment of risk of bias that may affect the cumulative evidence (e.g., publication bias, selective reporting within studies). | Page 7, paragraph 2; page 8, paragraph 1 and supplement 5 |  |  |
| Additional analyses | 16 | Describe methods of additional analyses (e.g., sensitivity or subgroup analyses, meta-regression), if done, indicating which were pre-specified. | Page 9, paragraph 1 |  |  |
| **RESULTS** | | |  |  |  |
| Study selection | 17 | Give numbers of studies screened, assessed for eligibility, and included in the review, with reasons for exclusions at each stage, ideally with a flow diagram. | Page 9, paragraph 2 and figure 1 |  |  |
| Study characteristics | 18 | For each study, present characteristics for which data were extracted (e.g., study size, PICOS, follow-up period) and provide the citations. | Page 9, paragraph 3; Page 10, paragraph 1; Table 1 and Tables 2 |  |  |
| Risk of bias within studies | 19 | Present data on risk of bias of each study and, if available, any outcome level assessment (see item 12). | Page 10, paragraph 2; Page 13, paragraph 1 and Figure S1b  Page 12, paragraph 2 and Figure S2b-5b  Page 13, paragraph 3; page 14 and paragraph 1 and Figure S6b-7b |  |  |
| Results of individual studies | 20 | For all outcomes considered (benefits or harms), present, for each study: (a) simple summary data for each intervention group (b) effect estimates and confidence intervals, ideally with a forest plot. | Figure 2-4 |  |  |
| Synthesis of results | 21 | Present results of each meta-analysis done, including confidence intervals and measures of consistency. | Page 11, paragraph 1-2; Page 12, paragraph 1; Page 13, paragraph 2 |  |  |
| Risk of bias across studies | 22 | Present results of any assessment of risk of bias across studies (see Item 15). | Page 10, paragraph 2; Page 13, paragraph 1 and Figure S1a  Page 12, paragraph 2 and Figure S2a-5a  Page 13, paragraph 3; page 14 and paragraph 1 and Figure S6a-7a |  |  |
| Additional analysis | 23 | Give results of additional analyses, if done (e.g., sensitivity or subgroup analyses, meta-regression [see Item 16]). | Table 3-5 |  |  |
| **DISCUSSION** | | |  |  |  |
| Summary of evidence | 24 | Summarize the main findings including the strength of evidence for each main outcome; consider their relevance to key groups (e.g., healthcare providers, users, and policy makers). | Page 14, paragraph 2 |  |  |
| Limitations | 25 | Discuss limitations at study and outcome level (e.g., risk of bias), and at review-level (e.g., incomplete retrieval of identified research, reporting bias). | Page 15, paragraph 2; Page 18, paragraph 2 |  |  |
| Conclusions | 26 | Provide a general interpretation of the results in the context of other evidence, and implications for future research. | Page 18, paragraph 3 and page 19, paragraph 1 |  |  |
| **FUNDING** | | |  |  |  |
| Funding | 27 | Describe sources of funding for the systematic review and other support (e.g., supply of data); role of funders for the systematic review. | Page 20, paragraph 2 |  |  |

**MOOSE checklist**

| **Reporting of background should include** | |
| --- | --- |
| Problem definition | Introduction, page 5, paragraph 1 |
| Hypothesis statement | Introduction, page 5, paragraph 3 |
| Description of study outcome(s) | Methods, page 6, paragraph 3; page 7, paragraph 1 and supplement 3 |
| Type of exposure or intervention used | Methods, page 6, paragraph 3 and supplement 3 |
| Type of study designs used | Methods, page 6, paragraph 3 and supplement 3 |
| Study population | General population including children and adult; Methods, page 6, paragraph 3 and supplement 3 |
| **Reporting of search strategy should include** | |
| Qualifications of searchers (eg, librarians and investigators) | Methods, page 6, paragraph 2 |
| Search strategy, including time period included in the synthesis and keywords | Supplement 2 |
| Effort to include all available studies, including contact with authors | Methods, page 7, paragraph 2; page 8, paragraph 3 |
| Databases and registries searched | Methods, page 6, paragraph 2 |
| Search software used, name and version, including special features used (eg, explosion) | Endnote X5 |
| Use of hand searching (eg, reference lists of obtained articles) | Methods, page 7, paragraph 1 |
| List of citations located and those excluded, including justification | Figure 1 |
| Method of addressing articles published in languages other than English | Methods, page 6, paragraph 2, searched CNKI (in Chinese) databases |
| Method of handling abstracts and unpublished studies | Figure 1 and Supplement 3 |
| Description of any contact with authors | page 8, paragraph 2 |
| **Reporting of methods should include** | |
| Description of relevance or appropriateness of studies assembled for assessing the hypothesis to be tested | Methods, page 6, paragraph 3 and supplement 3 |
| Rationale for the selection and coding of data (eg, sound clinical principles or convenience) | Methods, page 8, paragraph 2-3 |
| Documentation of how data were classified and coded (eg, multiple raters, blinding, and interrater reliability) | Methods, page 8, paragraph 3 |
| Assessment of confounding (eg, comparability of cases and controls in studies where appropriate) | Methods, page 9, paragraph 1, supplement 5 |
| Assessment of study quality, including blinding of quality assessors; stratification or regression on possible predictors of study results | Methods, page 7, paragraph 2, supplement 5 |
| Assessment of heterogeneity | Methods, page 9, paragraph 1 |
| Description of statistical methods (eg, complete description of fixed or random effects models, justification of whether the chosen models account for predictors of study results, dose-response models, or cumulative meta-analysis) in sufficient detail to be replicated | Methods, page 9, paragraph 1 |
| Provision of appropriate tables and graphics | Figure 1-4, S1-S7; Table 1-5 |
| **Reporting of results should include** | |
| Graphic summarizing individual study estimates and overall estimate | Figure 2-4, Table 3-5 |
| Table giving descriptive information for each study included | Table 1-2 |
| Results of sensitivity testing (eg, subgroup analysis) | Table 3-5 |
| Indication of statistical uncertainty of findings | We calculated 95% confidence intervals (CIs) of the pooled risk estimates (ORs or RRs) |
| **Reporting of discussion should include** | |
| Quantitative assessment of bias (eg, publication bias) | Discussion, page 12, paragraph 2; page 14, paragraph 1 |
| Justification for exclusion (eg, exclusion of non–English-language citations) | Discussion, page 18, paragraph 2 |
| Assessment of quality of included studies | Discussion, page 17, paragraph 2 |
| **Reporting of conclusions should include** | |
| Consideration of alternative explanations for observed results | No |
| Generalisation of the conclusions (ie, appropriate for the data presented and within the domain of the literature review) | Yes |
| Guidelines for future research | Conclusions, Page 18, paragraph 3 and page 19, paragraph 1 |
| Disclosure of funding source | Page 20, paragraph 2 |

**Supplement 2. Search strategy and results in Pubmed and Embase databases.**

**Search strategy in PubMed**

| **Search** | **Query** | **Items found** |
| --- | --- | --- |
| #1 | Search (((((((("Vitamin D"[Mesh]) OR Vitamin D[Text Word]) OR ("Calcitriol"[Mesh] OR Calcitriol[Text Word] OR "Receptors, Calcitriol"[Mesh]) OR (((1,25-dihydroxycholecalciferol[Text Word]) OR 1,25-dihydroxyvitamin D3[Text Word]) OR 1,25 AND (OH) AND 2D3[Text Word]) OR (("Cholecalciferol"[Mesh]) OR Cholecalciferol[Text Word]) OR (((1,25-Dihydroxyvitamin D[Text Word]) OR 25-Hydroxyvitamin D[Text Word]) OR 25-hydroxy vitamin D[Text Word]) OR (((25-Hydroxycholecalciferol[Text Word]) OR 1,25-Dihydroxycholecalciferol[Text Word]) OR 1 alpha, 25-dihydroxycholecalciferol[Text Word]) OR (("Vitamin D Deficiency"[Mesh]) OR Vitamin D Deficien*[Text Word]) OR (Vitamin D insufficien*[Text Word]))))))) Filters: Publication date to 2017/07/19 | [82615](https://www.ncbi.nlm.nih.gov/pubmed/?cmd=HistorySearch&querykey=2) |
| #2 | Search ((((((("Maternal Exposure"[Mesh]) OR maternal exposure[Text Word]) OR environmental exposure[MeSH Terms]) OR (((pregnancy[MeSH Terms]) OR pregnancy[Text Word]) OR Pregnanc*[Text Word]) OR (pregnant women[MeSH Terms] OR pregnant women[Text Word] OR pregnant woman[Text Word]) OR ((("Uterus"[Mesh]) OR Uter*[Text Word]) OR uterus[Text Word]) OR (("Fetus"[Mesh] OR Fetus*[Text Word]) OR Foetus[Text Word] OR Fetal[Text Word]) OR (((prenatal[Text Word] OR antenatal[Text Word])) OR Intrauterine[Text Word]) OR ((((("Fetal Blood"[Mesh]) OR fetal Blood*[Text Word]) OR foetale Blood*[Text Word]) OR cord Blood*[Text Word]) OR umbilical cord Blood*[Text Word]) OR (perinatal[Text Word]) OR (("Infant"[Mesh]) OR Infan*[Text Word]) OR (postpartum[Text Word]) OR gestation[Text Word])))) Filters: Publication date to 2017/07/19 | [2445338](https://www.ncbi.nlm.nih.gov/pubmed/?cmd=HistorySearch&querykey=3) |
| #3 | Search (((((((((((((((randomized controlled trial[Publication Type]) OR controlled clinical trial[Publication Type]) OR randomized[Title/Abstract]) OR placebo[Title/Abstract]) OR drug therapy[MeSH Subheading]) OR randomly[Title/Abstract]) OR trial[Title/Abstract]) OR groups[Title/Abstract])))) OR (((((risk*[Title/Abstract] OR risk*[MeSH:noexp] OR risk *[MeSH:noexp] OR cohort studies[MeSH Terms] OR group*[Text Word]) OR (odds[WORD] AND ratio*[ Text Word]) OR (case control*[Text Word] OR case-control studies[MeSH:noexp]))))))))) Filters: Publication date to 2017/07/19 | [7500381](https://www.ncbi.nlm.nih.gov/pubmed/?cmd=HistorySearch&querykey=4) |
| #4 | Search ((((((("Animals"[Mesh]) NOT ("Humans"[Mesh]) AND "Animals"[Mesh])))))) Filters: Publication date to 2017/07/19 | [4348407](https://www.ncbi.nlm.nih.gov/pubmed/?cmd=HistorySearch&querykey=5) |
| #5 | Search (#3) NOT #4 Filters: Publication date to 2017/07/19 | [6702229](https://www.ncbi.nlm.nih.gov/pubmed/?cmd=HistorySearch&querykey=6) |
| #6 | Search ((((((((food allergy[Text Word]) OR (((((((((("Allergy and Immunology"[Mesh]) OR "immunology"[Subheading])) OR immunology[Text Word]) OR Hypersensitivity[MeSH Terms]) OR Hypersensitivity[Text Word]))) OR allerg*[Text Word]) OR allergy[Text Word])) OR (allergic disease*[Text Word]) OR (((Bronchial Asthma[Text Word]) OR Asthma?[Text Word]) OR Asthma[MeSH Terms]) OR (((((((((((("Respiratory Sounds"[Mesh]) OR Respiratory Sound?[Text Word]) OR Breathing Sound?[Text Word]) OR Lung Sound?) OR Stridor?) OR Rale?) OR Crackle?) OR Pleural Rub?) OR Rhonchi) OR Rhonchus) OR Wheezing?) OR Wheez*) OR ((((Dermatiti*[Title/Abstract] AND Atopic[Title/Abstract]))) OR ((((((((Atopic Dermatitis[Text Word]) OR Atopic Dermatiti*[Text Word]) OR Atopic Neurodermatiti*[Text Word]) OR Disseminated Neurodermatiti*[Text Word]) OR Atopic Eczema[Text Word]) OR Infantile Eczema[Text Word]) OR eczema[Text Word]) OR "Dermatitis, Atopic"[Mesh])) OR ((((Atopic sensitization[Text Word]) OR sensitization[Text Word]) OR atopy[Text Word]) OR Atopic[Text Word]))))))) Filters: Publication date to 2017/07/19 | [1764964](https://www.ncbi.nlm.nih.gov/pubmed/?cmd=HistorySearch&querykey=7) |
| #7 | Search (#1 AND #2 AND #5 AND #6) Filters: Publication date to 2017/07/19 | [423](https://www.ncbi.nlm.nih.gov/pubmed/?cmd=HistorySearch&querykey=8) |

**Search strategy in Embase (1974 to 2014 December 09)**

| **Search** | **Query** | **Items found** |
| --- | --- | --- |
| #1 | exp vitamin D/ or vitamin d.mp. | 108656 |
| #2 | calcitriol.mp. or exp calcitriol derivative/ or exp calcitriol/ or exp calcitriol receptor/ | 30311 |
| #3 | (1,25-dihydroxycholecalciferol or 1,25-dihydroxyvitamin D3 or "1,25(OH)2D3").mp. | 11275 |
| #4 | (1,25-dihydroxyvitamin D or 25-Hydroxyvitamin D or 25-hydroxy vitamin D).mp. | 23356 |
| #5 | exp colecalciferol receptor/ or exp colecalciferol derivative/ or exp colecalciferol/ or colecalciferol.mp. | 54832 |
| #6 | (25-Hydroxycholecalciferol or 1,25-Dihydroxycholecalciferol or 1 alpha,25-dihydroxycholecalciferol).mp. | 2362 |
| #7 | vitamin d deficien$.mp. or exp vitamin D deficiency/ | 26870 |
| #8 | vitamin d insufficien$.mp. | 3408 |
| #9 | 1 or 2 or 3 or 4 or 5 or 6 or 7 or 8 | 138143 |
| #10 | maternal exposure.mp. or exp environmental exposure/ | 96840 |
| #11 | pregnancy.mp. or exp pregnancy/ or pregnan$.mp. | 898785 |
| #12 | pregnant women.mp. or exp pregnant woman/ or pregnant wom#n.mp. | 121878 |
| #13 | uterus.mp. or exp uterus/ or uter$.mp. | 407825 |
| #14 | exp fetus/ or (f?etus$ or f?etal).mp. | 459469 |
| #15 | prenatal.mp. or exp prenatal period/ or exp prenatal exposure/ or antenatal.mp. | 243734 |
| #16 | intrauterine.mp. | 91138 |
| #17 | f?etal blood.mp. or exp fetus blood/ | 11499 |
| #18 | cord blood.mp. or exp umbilical cord blood/ | 43673 |
| #19 | perinatal.mp. or exp perinatal period/ | 119316 |
| #20 | exp infant/ or infant$.mp. | 1134520 |
| #21 | postpartum.mp. | 59988 |
| #22 | 10 or 11 or 12 or 13 or 14 or 15 or 16 or 17 or 18 or 19 or 20 or 21 | 2493305 |
| #23 | exp risk/ or risk.mp. or exp risk factor/ or exp risk reduction/ or exp fetus risk/ or exp high risk pregnancy/ or exp high risk infant/ | 3010713 |
| #24 | cohort studies.mp. or exp cohort analysis/ | 26173 |
| #25 | Group.tw. | 3019344 |
| #26 | randomized controlled trial/ or controlled clinical trial/ or drug therapy/ or randomized.mp. or randomization/ or randomization.mp. or placebo.mp. or trial*.mp. or group*.mp. | 6252216 |
| #27 | risk/ or risk.mp. or odds ratio.mp. or cohort analysis/ or cohort stud*.mp. or case control study/ or case control.mp. or casecontrol.mp. | 3324895 |
| #28 | 23 or 24 or 25 or 26 or 27 | 8323758 |
| #29 | exp animal/ | 23390564 |
| #30 | exp human/ | 18715600 |
| #31 | 28 not 29 | 1155153 |
| #32 | 28 and 30 | 6343152 |
| #33 | 31 or 32 | 7498305 |
| #34 | 9 and 22 and 33 | 7348 |
| #35 | exp immunology/ or immunology.mp. | 427350 |
| #36 | delayed hypersensitivity.mp. or exp delayed hypersensitivity/ | 52084 |
| #37 | exp abnormal respiratory sound/ or exp lung auscultation/ or respiratory sounds.mp. or breathing sound.mp. or lung sound.mp. | 48323 |
| #38 | stridor$.mp. or exp stridor/ or rale$.mp. or crackle$.mp. or pleural rub$.mp. or rhonchi.mp. or rhonchus.mp. | 16072 |
| #39 | wheezing.mp. or exp wheezing/ or wheez$.mp. | 27256 |
| #40 | exp mild persistent asthma/ or exp extrinsic asthma/ or exp moderate persistent asthma/ or exp nocturnal asthma/ or exp intrinsic asthma/ or exp asthma/ or exp allergic asthma/ or exp severe persistent asthma/ or exp mild intermittent asthma/ or bronchial asthma.mp. or asthma$.mp. | 259925 |
| #41 | exp atopic dermatitis/ or exp sensitization/ or exp atopy/ or exp allergy/ or allergic disease/ or atopic dermatitis.mp. or atopic sensitization.mp. or sensiti$ation.mp. or (atopic or atopy).mp. | 182518 |
| #42 | (dermatiti$ or atopic dermatiti$ or atopic neurodermatiti$ or disseminated neurodermatiti$ or atopic eczema or infantile eczema or eczema).mp. or exp eczema/ or exp hand eczema/ | 133963 |
| #43 | allergic disease$.mp. or exp allergic disease/ | 22978 |
| #44 | exp food allergy/ or food allergy.mp. or exp hypersensitivity/ or allerg$.mp. or exp allergy/ or hypersensitivity.mp. | 641255 |
| #45 | 35 or 36 or 37 or 38 or 39 or 40 or 41 or 42 or 43 or 44 | 1158900 |
| #46 | 34 and 45 | 855 |

**Supplement 3. Form for guidance of title and abstract screening process.**

**Inclusion Criteria**

**Study design:**

- Prospective and retrospective cohort studies

- Randomized controlled trial, Quasi-randomized controlled trial, Non-randomized controlled trial

**Participants:**

General population including children and adult

**Exposure:**

-Antenatal vitamin D exposure: Maternal blood vitamin D level during any trimester of pregnancy, Cord blood vitamin D level, maternal intake of vitamin D during any trimester of pregnancy

-Vitamin D exposure during infancy: blood vitamin D level, intake of vitamin D

**Outcome:**

-Asthma

-Wheeze

**Exclusion criteria**

-Not published in English

-Conference abstract only

-Case-control, Cross-sectional, review, case report, and other uninterested study design

-Focused on measures of vitamin D exposure not during pregnancy or infancy

-Studies reporting other allergic disorders rather than asthma or wheeze

-Studies focused on the prognosis of asthma or wheeze

-Animal studies.

Please confirm eligibility for review in your user file (excel file):

1-yes

0-no

99-unsure

If the title or abstract is not clear enough to make a decision, please choose ‘unsure’ and a full text screening is needed.

Please also answer the following question in your user file.

Is it a related study that may be useful for the review?

1-yes

0-no

99-unsure

**Supplement 4. Form for guidance of full text assessment process.**

Decide on in- and exclusion of study based on the full text and the following inclusion criteria

| **Study ID** *(surname of first author and year first full report of study was published e.g. 1 Smith 2001)* | |
| --- | --- |
|  | |
| **Report IDs of other reports of this study** *(e.g. duplicate publications, follow-up studies)* |  |
|  |  |

| **Notes:** |
| --- |

## General Information

| **Date form completed** *(dd/mm/yyyy)* |  |
| --- | --- |
| **Name/ID of person extracting data** |  |
| **Report title**  *(title of paper/ abstract/ report that data are extracted from)* |  |
| **Report ID**  *(if there are multiple reports of this study)* |  |
| **Report author contact details** |  |
| **Publication type**  *(e.g. full report, abstract, letter)* |  |
| **Possible conflicts of interest**  *(for study authors)* |  |
| **Notes:** | |

## Eligibility

| **Study Characteristics** | **Review Inclusion Criteria**  *(Insert inclusion criteria for each characteristic as defined in the Protocol)* | | Yes | | No | Unclear | | |
| --- | --- | --- | --- | --- | --- | --- | --- | --- |
| **Type of study** | PCS=Prospective cohort study; | |  | |  |  | | |
|  | RCS=Retrospective cohort study | |  | |  |  | | |
|  | RCT=Randomized controlled trial; | |  | |  |  | | |
|  | Q-RCT=Quasi-randomized controlled trial; | |  | |  |  | | |
|  | NRCT=Non-randomized controlled trial | |  | |  |  | | |
|  | NCC=Nested case-control study; | |  | |  |  | | |
|  | CC=Case-control study | |  | |  |  | | |
|  | Other design (specify): | |  | |  |  | | |
| **exposures** | 1-maternal blood vitamin D level during pregnancy | |  | |  |  | | |
|  | 2- Cord blood vitamin D level | |  | |  |  | | |
|  | 3-Maternal vitamin D intake during pregnancy | |  | |  |  | | |
|  | 4-Infantile blood vitamin D level | |  | |  |  | | |
|  | 5-Infant vitamin D intake | |  | |  |  | | |
|  | Other (specify): | |  | |  |  | | |
| **outcomes** | 1-asthma | |  | |  |  | | |
|  | 2-wheeze | |  | |  |  | | |
|  | Other (specify): | |  | |  | |  | |
| **INCLUDE** | | **EXCLUDE** | | | | | | |
| **Reason for exclusion** | -not published in English | |  |  | |  | | |
|  | -review | |  |  | |  | | |
|  | -cross-sectionalor other uninterested study design | |  |  | |  | | |
|  | - focused on measures of vitamin D exposure not during pregnancy or infancy | |  |  | |  | | |
|  | - studies reporting other allergic disorders rather than asthma or wheeze | |  |  | |  | | |
|  | - studies focused on the prognosis of asthma or wheeze | |  |  | |  | | |
|  | - animal studies | |  |  | |  | | |
|  | - Other (specify): | |  |  | | | |  |
| **Notes:** | | | | | | | | |

DO NOT PROCEED IF STUDY EXCLUDED FROM REVIEW

**Supplement 5. Details on methods of data extraction and risk of bias assessment.**

The following items were recorded: study population, setting, inclusion criteria, exclusion criteria, method of recruitment of participants, informed consent obtained, aim of study, study design, Start date(period of enrollment), end date, duration of participation, interval of follow up (cohort studies and clinical trials only), exposures/intervention, exposure/intervention measurement, form of vitamin D measured, outcomes, outcome definition, outcome measurement, comparator(s)/ control, outcome collected time points, confounding factors, methods of dealt with confounding, number of participants identified, number of participants included in the analysis, number of subjects with and without asthma or wheeze in the antenatal or early postnatal vitamin D exposed/intervention and non-exposed/control groups; risk estimates (crude and/or adjusted odds ratios [ORs], relative risks [RRs] and hazard ratios [HRs], collectively referred to as odds ratios [ORs]) and the corresponding 95% confidence intervals (CIs) at any available age end point, other information such as key conclusions of study authors, miscellaneous comments from the study authors, references to other relevant studies, correspondence required for further study information.

Risk of  bias for each outcome within each study and across studies according study design was separately assessed using the tool recommended by the Cochrane collaboration. For cohort studies, we evaluated the selection of population, assessment of exposure, outcome and prognostic variables, whether outcome was present at the start of the study, whether prognostic variables were matched or adjusted for, whether follow-up was adequate, and whether co-intervention bias was present. For case-control studies, we evaluated exposure assessment, definition and selection of cases and controls, and whether prognostic variables were matched or adjusted for. The reviewers independently rated the risk of bias for each study as high, low, or unclear (‘Definitely yes’ in the tool was considered as ‘low’ risk, ‘Definitely no’ in the tool was considered as ‘high’ risk, ‘unclear’ comprised ‘probably yes’ and ‘probably no’ as specified in the tools.

For trials, we evaluated sequence generation, intervention allocation, blinding, whether follow-up was adequate, whether there was selective reporting of outcome, and other sources of bias.

Tool to Assess Risk of Bias in Cohort Studies

| Domain | Risk of bias | | | | Examples |
| --- | --- | --- | --- | --- | --- |
|  | Definitely yes | Probably yes | Probably no | Definitely no |  |
| 1. Was selection of exposed and non-exposed cohorts drawn from the same population? |  |  |  |  | Examples of low risk of bias: Exposed and unexposed drawn for same administrative data base of patients presenting at same points of care over the same time frame  Examples of high risk of bias: exposed and unexposed presenting to different points of care or over a different time frame |
| 2. Can we be confident in the assessment of exposure? |  |  |  |  | Examples of low risk of bias: Secure record [e.g. surgical records, pharmacy records]; Repeated interview or other ascertainment asking about current use/exposure  Examples of higher risk of bias: Structured interview at a single point in time; Written self report; Individuals who are asked to retrospectively confirm their exposure status may be subject to recall bias – less likely to recall an exposure if they have not developed an adverse outcome, and more likely to recall an exposure (whether an exposure occurred or not) if they have developed an adverse outcome.  Examples of high risk of bias: uncertain how exposure information obtained |
| 3. Can we be confident that the outcome of interest was not present at start of study |  |  |  |  |  |
| 4. Did the study match exposed and unexposed for all variables that are associated with the outcome of interest or did the statistical analysis adjust for these prognostic variables? |  |  |  |  | Examples of low risk of bias: comprehensive matching or adjustment for all plausible prognostic variables, for example: Age, sex, ethnicity and socio-economic status (SES), family history of atopic diseases, seasonal variations, tobacco smoking, maternal body mass index, sedentary lifestyle, breastfeeding, ect.  Examples of higher risk of bias: matching or adjustment for most plausible prognostic variables  Examples of high risk of bias: matching or adjustment for a minority of plausible prognostic variables, or no matching or adjustment at all. Statements of no differences between groups or that differences were not statistically significant are not sufficient for establishing comparability. |
| 5. Can we be confident in the assessment of the presence or absence of prognostic factors? |  |  |  |  | Examples of low risk of bias: Interview of all participants; self-completed survey from all participants; review of charts with reproducibility demonstrated; from data base with documentation of accuracy of abstraction of prognostic data  Examples of higher risk of bias: Chart review without demonstration of reproducibility; data base with uncertain quality of abstraction of prognostic information  Examples of high risk of bias: Prognostic information from data base with no available documentation of quality of abstraction of prognostic variables |
| 6. Can we be confident in the assessment of outcome? |  |  |  |  | Examples of low risk of bias: Independent blind assessment; Record linkage; For some outcomes (e.g. fractured hip), reference to the medical record is sufficient to satisfy the requirement for confirmation of the fracture.  Examples of higher risk of bias: Independent assessment unblinded; self-report; For some outcomes (e.g. vertebral fracture where reference to x-rays would be required) reference to the medical record would not be adequate outcomes.  Examples of high risk of bias: uncertain (no description) |
| **7. Was the follow up of cohorts adequate?** |  |  |  |  | Examples of low risk of bias: No missing outcome data; Reasons for missing outcome data unlikely to be related to true outcome (for survival data, censoring is unlikely to introduce bias); Missing outcome data balanced in numbers across intervention groups, with similar reasons for missing data across groups; For dichotomous outcome data, the proportion of missing outcomes compared with observed event risk is not enough to have a important impact on the intervention effect estimate; For continuous outcome data, plausible effect size (difference in means or standardized difference in means) among missing outcomes is not large enough to have an important impact on the observed effect size; Missing data have been imputed using appropriate methods.  Examples of high risk of bias: Reason for missing outcome data likely to be related to true outcome, with either imbalance in numbers or reasons for missing data across intervention groups; For dichotomous outcome data, the proportion of missing outcomes compared with observed event risk is enough to induce important bias in intervention effect estimate; For continuous outcome data, plausible effect size (difference in means or standardized difference in means) among missing outcomes is large enough to induce clinically relevant bias in the observed effect size; Potentially inappropriate application of simple imputation. |
| **8. Were co-Interventions similar between groups?** |  |  |  |  | Examples of low risk of bias: Most or all relevant co-interventions that might influence the outcome of interest are documented to be similar in the exposed and unexposed.  Examples of high risk of bias: Few or no relevant co-interventions that might influence the outcome of interest are documented to be similar in the exposed and unexposed. |

**Table S1 Characteristics of studies excluded by full-text screening.**

| **Study** | **Type of study** | **exposures** | **outcomes** | **Reason for exclusion** |
| --- | --- | --- | --- | --- |
| Weisse 2013[[1](#_ENREF_1)] | PCS | Maternal vitamin D level during pregnancy Cord blood vitamin D level | Food allergy | outcome not relevant |
| Liu 2013[[2](#_ENREF_2)] | PCS | Cord blood vitamin D level Blood 25(OH)D measured at 0–3 y | Food sensitization | outcome not relevant |
| Liu 2011[[3](#_ENREF_3)] | PCS | Cord blood vitamin D level | Food allergy | outcome not relevant |
| Nwaru 2010[[4](#_ENREF_4)] | PCS | maternal intake of vitamin D during pregnancy | Atopic sensitization | outcome not relevant |
| Kull 2006[[5](#_ENREF_5)] | PCS | vitamins A and D，in water-soluble form vs in peanut oil | Atopic sensitization | exposure not relevant |
| Hibbs 2014[[6](#_ENREF_6)] | PCS | Use of multivitamins | Wheeze | exposure not relevant |
| Gazibara 2015[[7](#_ENREF_7)] | PCS | Maternal vitamin D level during pregnancy Cord blood vitamin D level | Atopic dermatitis (eczema) | outcome not relevant |
| Jones 2015[[8](#_ENREF_8)] | PCS | Cord blood vitamin D level | Atopic dermatitis (eczema) | outcome not relevant |
| Koplin 2015[[9](#_ENREF_9)] | PCS | Vitamin D level during infancy | Food allergy | outcome not relevant |
| Grant 2016[[10](#_ENREF_10)] | RCT | Maternal vitamin D supplements during pregnancy  Vitamin D supplements during infancy | Food allergy | outcome not relevant |
| Molloy 2017[[11](#_ENREF_11)] | PCS | Cord blood vitamin D level Vitamin D level during infancy | Atopic dermatitis (eczema) Food allergy | outcome not relevant |
| Savilahti 2016[[12](#_ENREF_12)] | PCS | Cord blood vitamin D level | Atopic dermatitis (eczema)  Atopic sensitization  Allergic disorders | outcome not relevant |
| Tuokkola 2016[[13](#_ENREF_13)] | PCS | Maternal vitamin D supplements during pregnancy | Food allergy | outcome not relevant |
| Berents 2016[[14](#_ENREF_14)] | PCS | Vitamin D level during infancy | Atopic dermatitis (eczema) | outcome not relevant |
| Bunyavanich 2016[[15](#_ENREF_15)] | PCS | Maternal vitamin D level during pregnancy | Allergic rhinitis | outcome not relevant |
| Wu 2017 (in Chinese)[[16](#_ENREF_16)] | PCS | Cord blood vitamin D level | **Atopic sensitization** | outcome not relevant |

PCS=Prospective cohort study; RCT=Randomized controlled trial; NRCT=Non-randomized controlled trial;

**Table S2 The Number Needed to Treat estimate for preventing asthma by increasing from low to high levels of vitamin D or via supplementation**

|  | Asthma prevalence | Pooled OR | | | Number Needed to Treat | | |
| --- | --- | --- | --- | --- | --- | --- | --- |
|  |  | Point estimate | Lower limit | Upper limit | Point estimate | Lower limit | Upper limit |
| **Blood, age >5 y** |  |  |  |  |  |  |  |
| Mean prevalence | 9.4% | 0.95 | 0.69 | 1.31 | 213 | 35 | -35 |
| Min prevalence | 4.0% | 0.95 | 0.69 | 1.31 | 500 | 81 | -81 |
| Max prevalence | 16.0% | 0.95 | 0.69 | 1.31 | 125 | 21 | -21 |
| **Blood, age≤5 y** |  |  |  |  |  |  |  |
| Mean prevalence | 13.5% | 0.81 | 0.65 | 1.01 | 40 | 22 | -742 |
| Min prevalence | 4% | 0.81 | 0.65 | 1.01 | 132 | 72 | -2500 |
| Max prevalence | 31.2% | 0.81 | 0.65 | 1.01 | 17 | 10 | -321 |
| **Intake, age >5 y** |  |  |  |  |  |  |  |
| Mean prevalence | 9.4% | 0.73 | 0.56 | 0.94 | 39 | 24 | 177 |
| Min prevalence | 4% | 0.73 | 0.56 | 0.94 | 93 | 57 | 417 |
| Max prevalence | 14.8% | 0.73 | 0.56 | 0.94 | 25 | 15 | 113 |
| **Intake, age≤5 y** |  |  |  |  |  |  |  |
| Mean prevalence | 8.6% | 0.85 | 0.7 | 1.04 | 78 | 39 | -292 |
| Min prevalence | 4.1% | 0.85 | 0.7 | 1.04 | 163 | 81 | -610 |
| Max prevalence | 11.7% | 0.85 | 0.7 | 1.04 | 57 | 28 | -214 |

**Table S3 Publication bias of the cohort studies included by exposure and outcome**

|  |  | | |
| --- | --- | --- | --- |
|  | Bias | p | Funnel plots |
| **Vitamin D in blood and asthma>5 y** | 0.051 | 0.963 | 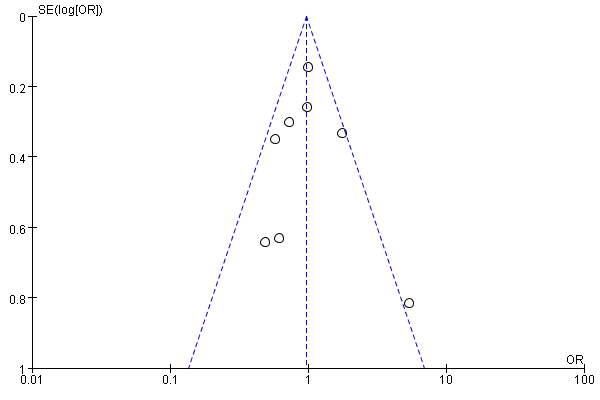 |
| **Vitamin D in blood and asthma≤5 y** | 0.435 | 0.655 | 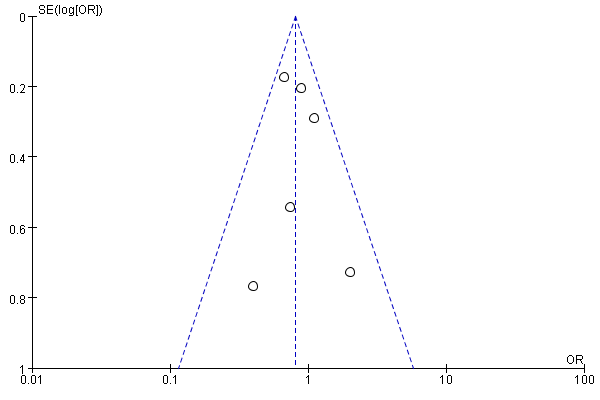 |
| **Vitamin D in blood and wheeze** | -0.407 | 0.772 | 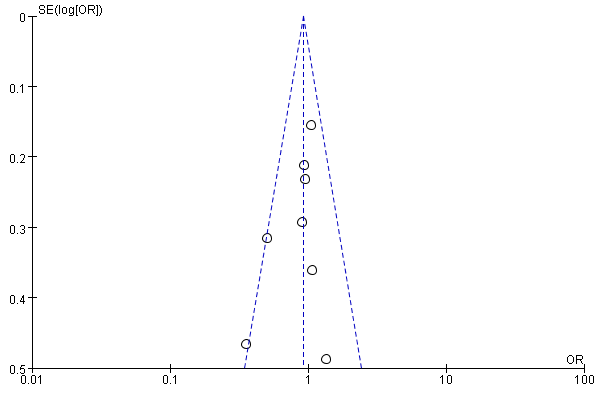 |
| **Vitamin D intake and asthma>5y** | -0.623 | 0.913 | 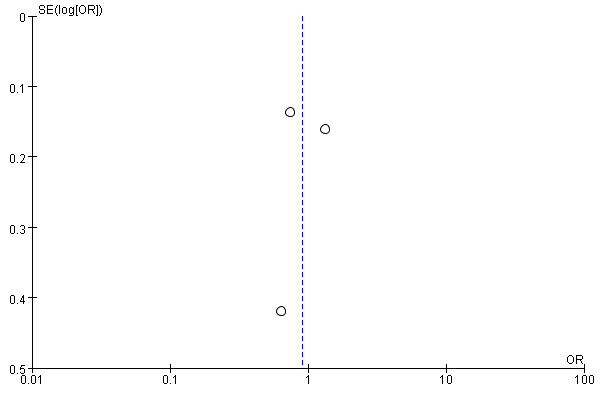 |
| **Vitamin D intake and asthma≤5y** | -0.876 | 0.410 | 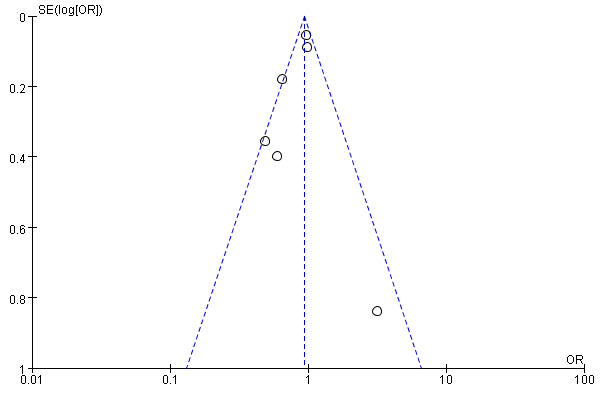 |
| **Vitamin D intake and wheeze** | -0.106 | 0.954 | 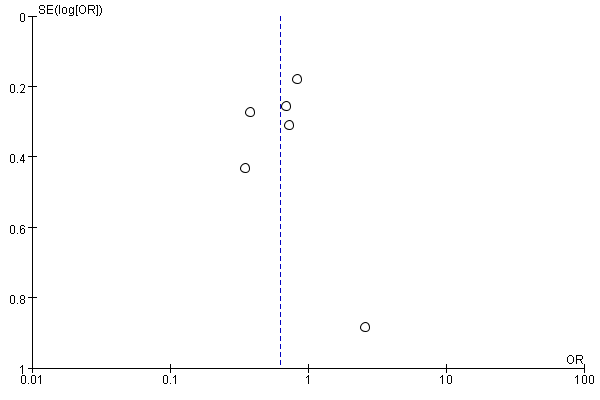 |


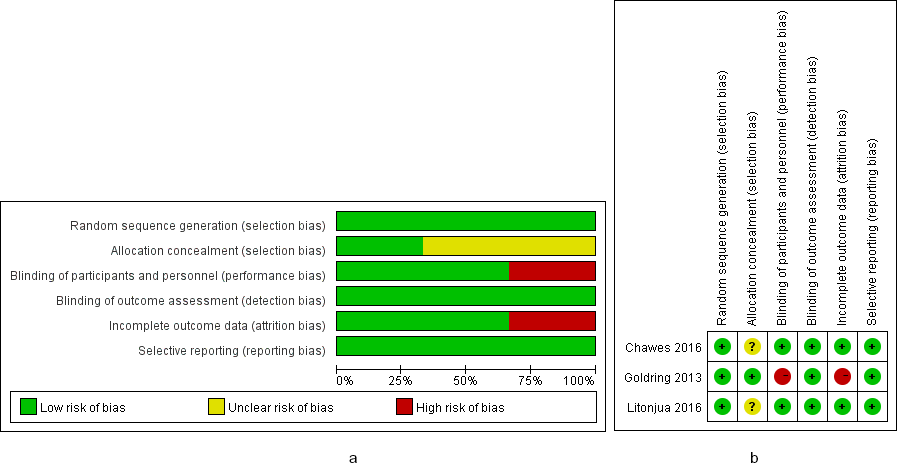


**Figure S1 Risk of bias for randomized controlled trials on maternal vitamin D supplementation and offspring asthma/wheeze**

**
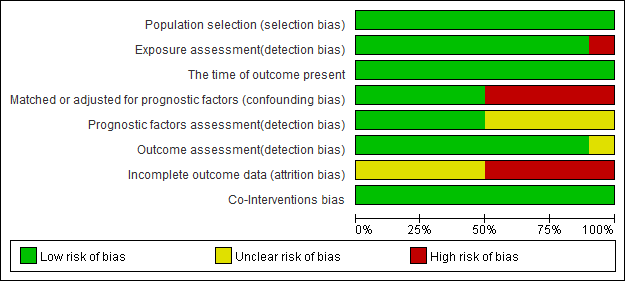

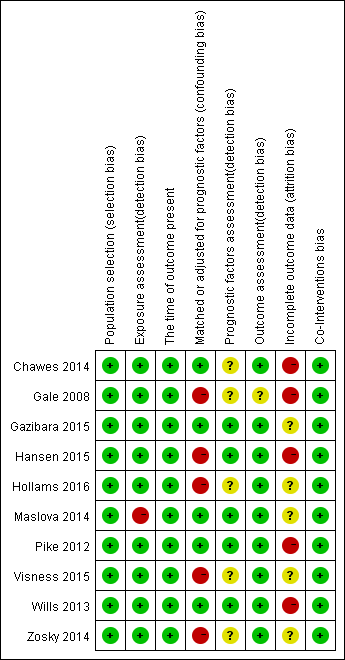
**

a b

**Figure S2 Risk of bias for cohort studies on early life blood vitamin D levels and asthma assessed >5 years.**

**
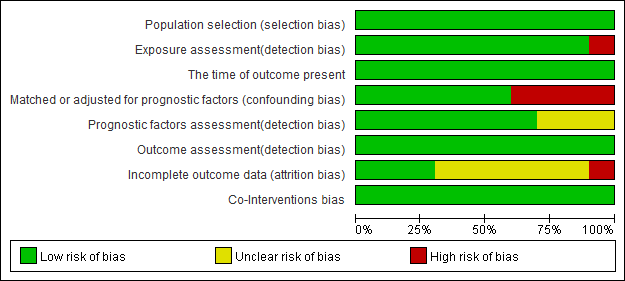

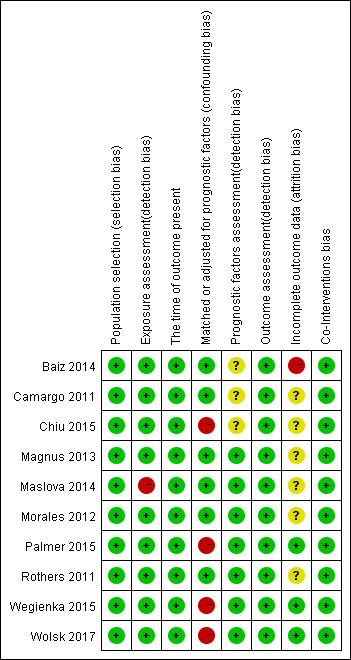
**

a b

**Figure S3 Risk of bias for cohort studies on early life blood vitamin D levels and asthma assessed** ≤**5 years.**

**
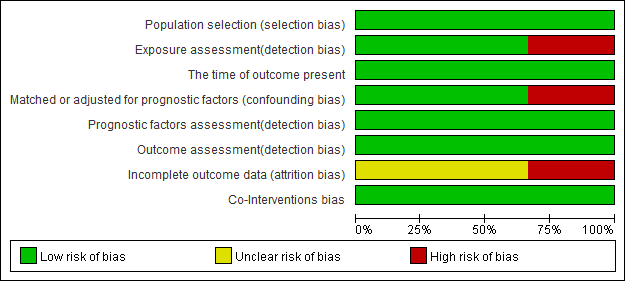

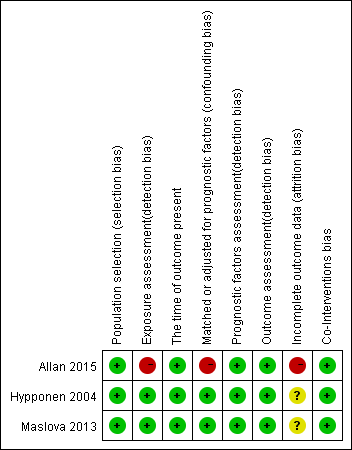
**

a b

**Figure S4 Risk of bias for cohort studies on early life vitamin D intake and asthma assessed >5 years.**

**
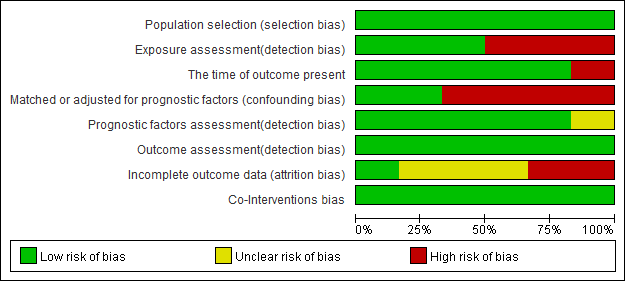

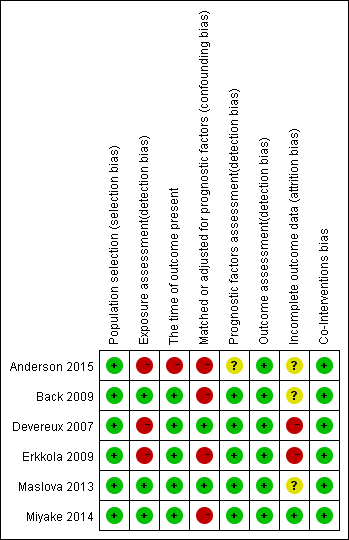
**

a b

**Figure S5 Risk of bias for cohort studies on early life vitamin D intake and asthma assessed** ≤**5 years.**

**
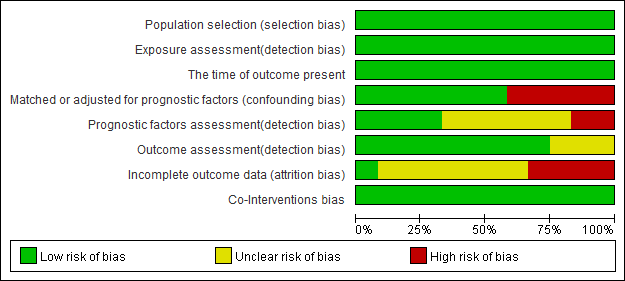

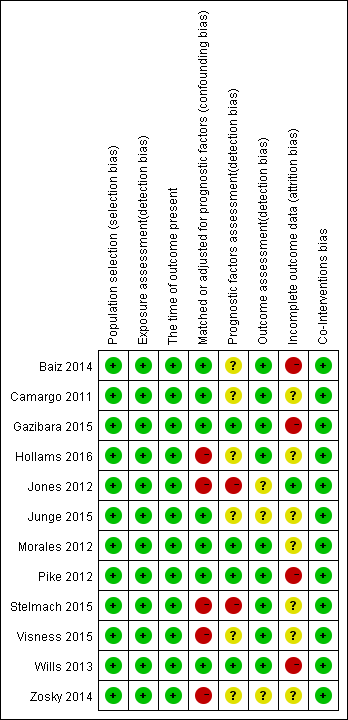
**

a b

**Figure S6 Risk of bias for cohort studies on early life blood vitamin D level and wheeze.**

**
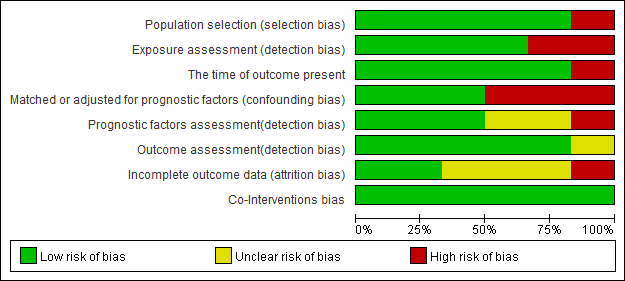
** **
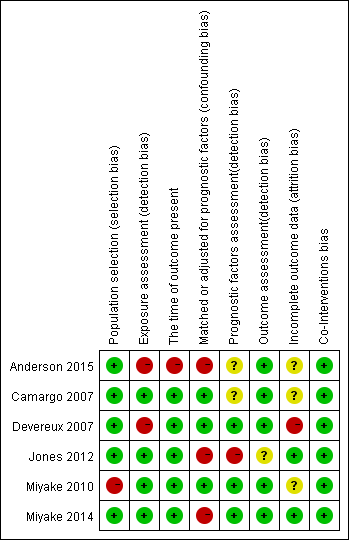
**

a b

**Figure S7 Risk of bias of cohort studies on early life vitamin D intake and wheeze**

**Reference**

**1. Weisse K, Winkler S, Hirche F, Herberth G, Hinz D, Bauer M, Roder S, Rolle-Kampczyk U, Von Bergen M, Olek S *et al*: Maternal and newborn vitamin D status and its impact on food allergy development in the German LINA cohort study. *Allergy: European Journal of Allergy and Clinical Immunology* 2013, 68(2):220-228.**

**2. Liu X, Arguelles L, Zhou Y, Wang G, Chen Q, Tsai HJ, Hong X, Liu R, Price HE, Pearson C *et al*: Longitudinal trajectory of vitamin D status from birth to early childhood in The development of food sensitization. *Pediatric Research* 2013, 74(3):321-326.**

**3. Liu X, Wang G, Hong X, Wang D, Tsai HJ, Zhang S, Arguelles L, Kumar R, Wang H, Liu R *et al*: Gene-vitamin D interactions on food sensitization: a prospective birth cohort study. *Allergy* 2011, 66(11):1442-1448.**

**4. Nwaru BI, Ahonen S, Kaila M, Erkkola M, Haapala AM, Kronberg-Kippila C, Veijola R, Ilonen J, Simell O, Knip M *et al*: Maternal diet during pregnancy and allergic sensitization in the offspring by 5 yrs of age: A prospective cohort study. *Pediatric Allergy and Immunology* 2010, 21(1 PART I):29-37.**

**5. Kull I, Bergstrom A, Melen E, Lilja G, van Hage M, Pershagen G, Wickman M: Early-life supplementation of vitamins A and D, in water-soluble form or in peanut oil, and allergic diseases during childhood. *The Journal of allergy and clinical immunology* 2006, 118(6):1299-1304.**

**6. Hibbs AM, Babineau DC, Wang X, Redline S: Race differences in the association between multivitamin exposure and wheezing in preterm infants. *Journal of perinatology : official journal of the California Perinatal Association* 2015, 35(3):192-197.**

**7. Gazibara T, Elbert NJ, den Dekker HT, de Jongste JC, Reiss I, McGrath JJ, Eyles DW, Burne TH, Tiemeier H, Jaddoe VW *et al*: Associations of maternal and fetal 25-hydroxyvitamin D levels with childhood eczema. The Generation R Study. *Pediatric allergy and immunology : official publication of the European Society of Pediatric Allergy and Immunology* 2015.**

**8. Jones AP, D'Vaz N, Meldrum S, Palmer DJ, Zhang G, Prescott SL: 25-hydroxyvitamin D3 status is associated with developing adaptive and innate immune responses in the first 6 months of life. *Clinical and experimental allergy : journal of the British Society for Allergy and Clinical Immunology* 2015, 45(1):220-231.**

**9. Koplin JJ, Suaini NH, Vuillermin P, Ellis JA, Panjari M, Ponsonby AL, Peters RL, Matheson MC, Martino D, Dang T *et al*: Polymorphisms affecting vitamin D-binding protein modify the relationship between serum vitamin D (25[OH]D) and food allergy. *The Journal of allergy and clinical immunology* 2015, 137(2):500-506.**

**10. Grant CC, Crane J, Mitchell EA, Sinclair J, Stewart A, Milne T, Knight J, Gilchrist C, Camargo CA, Jr.: Vitamin D supplementation during pregnancy and infancy reduces aeroallergen sensitization: a randomized controlled trial. *Allergy* 2016, 71(9):1325-1334.**

**11. Molloy J, Koplin JJ, Allen KJ, Tang MLK, Collier F, Carlin JB, Saffery R, Burgner D, Ranganathan S, Dwyer T *et al*: Vitamin D insufficiency in the first 6 months of infancy and challenge-proven IgE-mediated food allergy at 1 year of age: a case-cohort study. *Allergy* 2017, 72(8):1222-1231.**

**12. Savilahti EM, Makitie O, Kukkonen AK, Andersson S, Viljakainen H, Savilahti E, Kuitunen M: Serum 25-Hydroxyvitamin D in Early Childhood Is Nonlinearly Associated with Allergy. *International archives of allergy and immunology* 2016, 170(3):141-148.**

**13. Tuokkola J, Luukkainen P, Kaila M, Takkinen HM, Niinisto S, Veijola R, Virta LJ, Knip M, Simell O, Ilonen J *et al*: Maternal dietary folate, folic acid and vitamin D intakes during pregnancy and lactation and the risk of cows' milk allergy in the offspring. *The British journal of nutrition* 2016, 116(4):710-718.**

**14. Berents TL, Lodrup Carlsen KC, Mowinckel P, Sandvik L, Skjerven HO, Rolfsjord LB, Kvenshagen B, Hunderi JO, Bradley M, Lieden A *et al*: Vitamin D levels and atopic eczema in infancy and early childhood in Norway: a cohort study. *The British journal of dermatology* 2016, 175(1):95-101.**

**15. Bunyavanich S, Rifas-Shiman SL, Platts-Mills TA, Workman L, Sordillo JE, Camargo CA, Jr., Gillman MW, Gold DR, Litonjua AA: Prenatal, perinatal, and childhood vitamin D exposure and their association with childhood allergic rhinitis and allergic sensitization. *The Journal of allergy and clinical immunology* 2016, 137(4):1063-1070 e1061-1062.**

**16. Wu CK, Xia Y, Cao YB, Ji RF, Fu B, Sun PL: Correlation study on newborn cord blood 25(OH)D and IgE levels. *Chinese Journal of Child Health Care* 2017, 25(2):124-127 (in Chinese).**
